# Global Circulation Dynamics and Its Determinants of Dengue Virus: A Network Evolution and Model Study from 1990 to 2019

**DOI:** 10.3390/v17081078

**Published:** 2025-08-04

**Authors:** Haoyu Long, Jinfeng Zeng, Yilin Chen, Kang Tang, Chi Zhang, Qianru Sun, Lei Gao, Yuhui Lin, Junting He, Chunhui Yang, Xiaoying Lin, Wenzhe Su, Kuibiao Li, Biao Di, Min Kang, Chongguang Yang, Xiangjun Du

**Affiliations:** 1 School of Public Health (Shenzhen), Sun Yat-sen University, Guangzhou 510275, China; longhy7@mail2.sysu.edu.cn (H.L.); zengjf7@mail2.sysu.edu.cn (J.Z.); chenylin78@mail2.sysu.edu.cn (Y.C.); tangk25@mail2.sysu.edu.cn (K.T.); zhangch295@mail2.sysu.edu.cn (C.Z.); sunqr7@mail2.sysu.edu.cn (Q.S.); gaolei101@outlook.com (L.G.); linyh75@mail2.sysu.edu.cn (Y.L.); hejt7@mail2.sysu.edu.cn (J.H.); yangchh28@mail2.sysu.edu.cn (C.Y.); linxy96@mail2.sysu.edu.cn (X.L.); yangchg9@mail.sysu.edu.cn (C.Y.); 2 School of Public Health (Shenzhen), Shenzhen Campus of Sun Yat-sen University, Shenzhen 518107, China; 3 Key Laboratory of Public Health Safety, School of Public Health, Ministry of Education, Fudan University, Shanghai 200032, China; 4 School of Biomedical Engineering, Guangdong Medical University, Dongguan 523808, China; 5 School of Public Health, Guangdong Medical University, Dongguan 523808, China; 6 Preventive Health Division, Xijing Hospital, Air Force Medical University (The Fourth Military Medical University), Xi’an 710032, China; 7 Guangzhou Center for Disease Control and Prevention, Guangzhou 510440, China; suwenzhe@163.com (W.S.); kuibiao@outlook.com (K.L.); biao65di@yahoo.com (B.D.); 8 Guangdong Provincial Center for Disease Control and Prevention, Guangzhou 511430, China; kangmin@yeah.net; 9 Shenzhen Key Laboratory of Pathogenic Microbes & Biosecurity, Shenzhen Campus of Sun Yat-sen University, Shenzhen 518107, China; 10 Key Laboratory of Tropical Disease Control, Ministry of Education, Sun Yat-sen University, Guangzhou 510030, China

**Keywords:** dengue virus, global circulation, mutation network, circulation indicators, influencing factors, persistence

## Abstract

As dengue is an increasing global health threat, a better understanding of the global circulation dynamics and its determinants would be helpful for precise prevention and control of dengue. The dynamics of global circulation of the four dengue virus serotypes were explored utilizing genetic sequences through a network-based method. Four new circulation indicators, including local intensity, betweenness centrality, tip frequency, and persistence time, were defined. Three circulation roles, including source, hub, and destination, were proposed on the basis of new indicators. Spatial and temporal changes of the three circulation roles, along with the persistence time, were explored. Important determinants were also evaluated by machine learning models. Thailand, Indonesia, and Vietnam in Asia and Venezuela and Colombia in Americas were the sources for all four serotypes in different decades. Destinations were observed mostly in island regions. Over the decades, the number of regions with different circulation roles and persistence of DENV-1 increased significantly. Climate and airline factors were involved in the important determinants to circulation roles and persistence of dengue. The roles identified in the global circulation of dengue and important determinants, including climate and airline factors, provide new insights into global dynamics and are beneficial for controlling dengue.

## 1. Introduction

Dengue is a vector-borne infectious disease caused by dengue virus (DENV). The dominant vectors capable of transmitting the dengue virus are *Aedes aegypti* and *Aedes albopictus* [1]. Dengue virus is a single positive-stranded RNA virus, which can be categorized into four serotypes, including DENV-1, DENV-2, DENV-3, and DENV-4 [2]. Dengue virus has an about 10.7 kb of RNA genomes encoding structural (C, prM, E) and non-structural proteins (NS1, NS2A, NS2B, NS3, NS4A, NS4B, NS5). The envelope (E) protein is crucial for viral entry, immune response, and serotype classification and it has sufficient sequence diversity to allow phylogenetic analysis and strain diversity, making it ideal for molecular epidemiology [3].

It is estimated that there are approximately 390 million dengue infections and approximately 10,000 dengue deaths per year [4,5]. Infections with dengue virus can cause cases that are clinically mild or asymptomatic, symptomatic, and even severe symptomatic, such as vascular leakage, shock, and organ failure [6], especially secondary infections of heterogeneous serotypes due to antibody-dependent enhancement [7,8]. Unfortunately, there is currently no effective anti-dengue drug [9] and the effectiveness of the vaccine depends on serostatus and to date, no dengue vaccine is widely used [10,11].

With climate change, urbanization, and the increase in human movement around the world, dengue has expanded worldwide [12,13]. Many studies focus on the spread of the dengue virus to a particular region or serotype [14,15,16,17], while some studies focus on the spread of dengue to a continental scale [18,19,20]. However, there is less systematic research on global dengue circulation, particularly comparative studies of all four serotypes, making it difficult for us to have a comprehensive understanding of the dynamics of global dengue virus circulation.

A study on dengue transmission in Asia found that Thailand and India can play a role as sources of dengue in Asia, and Singapore and China as hubs [19]. But their roles in the global circulation of dengue are not clear. Furthermore, the circulation of dengue virus is a dynamic and complex process, so it is important to identify the role of the circulation at both spatial and temporal levels. In the context of the global spread of dengue, another problem that deserves our attention is the persistence of the dengue virus in some regions [21,22,23]. There is no doubt that the persistence of the dengue virus will be an important obstacle to the elimination of the dengue virus. However, there is a lack of quantitative studies that systematically clarify the pattern of persistence of dengue virus. In addition, climate, socioeconomic factors, and population factors have been shown to be associated with the spread of dengue in many studies [13,24,25,26]. However, these studies consider the number of cases or estimates of dengue as dependent variables. It is not yet clear whether other important indicators for the global dynamics of dengue, and not to mention the specific factors behind the cause, are important for accurate and targeted prevention and control of dengue.

In fact, due to limitations in the current surveillance system and efforts [27], there is a lack of epidemiological data specific to the serotype of the disease, which certainly obstructs the study of the distribution dynamics of the disease. Fortunately, the scale of genetic evolution of RNA viruses is often consistent with their transmission and epidemics [28], presenting another way to study the virus in detail [18,29,30,31]. First, phylogenetic trees are widely used to explore the transmission of different types of viruses. However, network-based methods have been developed to study the efficient transmission of infectious viruses [32,33], providing new ways of investigating the global spread of dengue based on genetic sequences. In addition, new technologies have expanded the scope of research with the SHapley Additive exPlanation framework (SHAP) developed and applied to interpret contributions and interactions of various factors based on a machine learning model [34,35]. Thus, although the process of dengue movement may be complex and influenced by several factors, important factors and their quantitative contributions can now be explored.

Therefore, although the phylogenetic tree is a good way, the study here is an attempt to provide some new insights into the global circulation of dengue based on a network-based approach using DENV E gene sequences. Four circulation indicators were defined, including local intensity, betweenness centrality, tip frequency, and persistence time, and were used to study the global circulation dynamics of dengue from 1990 to 2019. Three circulation roles, including source, hub, and destination, were also proposed to describe the global circulation of dengue. In addition, spatial and temporal changes in circulation roles and persistence time were investigated to provide a systematic overview of the dynamic changes in the global circulation of dengue over the past three decades. In addition, related factors were identified to provide targeted information for precise intervention in the control of dengue.

## 2. Methods

### 2.1. Data Collection and Processing

#### 2.1.1. Sequence Data

The E gene sequences of four serotypes of dengue viruses were collected from Virus Variation by the National Center for Biotechnology Information (NCBI) [36]. First, those who did not have time and spatial information were removed. Subsequently, the specific sequences of the serotype were aligned separately using Muscle v3.8 with the default settings [37]. Then, according to the same year and region, duplicate sequences were eliminated. Finally, there were a total of 4396 DENV-1, 2794 DENV-2, 1530 DENV-3, and 978 DENV-4 sequences. The time coverage of the sequence data is from the earliest (DENV-1, 2:1944, DENV-3, 4:1956) to 2019, and more than 70 countries or regions around the world were included. The number of countries or regions with four serotypes sequences available over three decades, including 1990–1999 (1990s), 2000–2009 (2000s), and 2010–2019 (2010s), is shown in Table S1.

#### 2.1.2. Case Data

Annual data on dengue cases in countries or regions were derived from the WHO Dengue Data Application (https://ntdhq.shinyapps.io/dengue5/ accessed on 23 June 2022). Dengue cases include clinically suspected and laboratory-confirmed cases. These data cover 120 countries or regions from 1990 to 2018. Furthermore, given the scope of this study to 2019. Therefore, the average number of cases from 1990 to 2018 for each country or region was used to represent cases in 2019. In addition, data on dengue cases in Taiwan region were collected by the Taiwan Center for Disease Control.

#### 2.1.3. Socioeconomic Data

Annual socioeconomic data of these countries or regions were collected from the World Bank (https://databank.worldbank.org/reports.aspx?source=World-Development-Indicators accessed on 22 March 2022). Socioeconomic data include GDP per capita (*GDP*), physicians per 1000 people (*physicians*), and secondary school enrollment (*school enrollment*). Socioeconomic data are available between 1960 and 2020. The detailed description of these variables is shown in Table S2. The missing data were filled through linear interpolation.

#### 2.1.4. Population Data

Annual population data of the countries or regions were also collected from the World Bank (https://databank.worldbank.org/reports.aspx?source=World-Development-Indicators accessed on 22 March 2022). Population data include population density, urban population, rural population, and urban population growth. Population data are available from 1960 to 2020. The detailed description of these variables is shown in Table S2. The missing data were filled by linear interpolation.

#### 2.1.5. Forest Data

In addition, the coverage of forest area (*forest area*) was used in this study to represent the ecological effects, and the data were also obtained from the World Bank (https://databank.worldbank.org/reports.aspx?source=World-Development-Indicators accessed on 22 March 2022). Annual forest data are available from 1960 to 2020. The detailed description is in Table S2. The missing data were filled by linear interpolation.

#### 2.1.6. Climate Data

Climate data include diurnal temperature range, cloud cover, ground frost frequency, potential evapotranspiration, precipitation, mean temperature, maximum temperature, minimum temperature, rain days, and vapor pressure. The annual average values of the above variables were collected from the CRU CY4.05 dataset [38]. Then, the relative humidity was calculated by the empirical formula recommended by Emanuel on the basis of vapor pressure and mean temperature [39]. Climate data are available from 1901 to 2020. The detailed description of these variables is shown in Table S3.

#### 2.1.7. Airline Data

Airline passenger flow data were collected from the Official Aviation Guide (https://analytics.oag.com/analyser-client/home accessed on 23 June 2022). First, airline passengers were separated into inbound, outbound passengers and domestic parts. To be simple, the inbound and outbound passengers were combined into the variable of external air passenger flow, and the domestic passenger was the internal passenger for each specific region. Furthermore, considering the incidence rate in the different departure regions, three variables were calculated, including cases exported by air, cases imported by air, and intra-regional cases by air and the detailed description of these variables is shown in Table S4. Annual data on airline passenger flows are available from 2011 to 2019.

### 2.2. Mutation Network Construction

To explore the dynamics of the circulation of dengue virus, mutation networks were dynamically constructed in three stages. The network method was generated and verified in detail in the previous study [33]. In particular, the network in the first stage was built on the sequences from the early year to 1999. The second and third stages were the earliest years until 2009 and 2019, respectively. The networks were constructed for the four serotypes separately and the network in each stage was constructed in the following way.

#### 2.2.1. Sampling Based on the Dengue Cases

Given that the sample size of the sequences is uneven in different regions for different reasons, there may be biases that deviate from the real situation. To coincide with the scale of dengue epidemics in different regions, the number of cases of dengue was taken into account during the sampling of sequences and simulated the actual situation of the dengue epidemic to some extent. For the regions where annual case data were available, the expected number of sequences per year was calculated based on the proportion of dengue cases per region among all cases within the regions for the year.

(1) $$N_{i}=S_{i}\times \frac{C_{i}}{\sum C}$$

For a given year, $N_{i}$ indicates the expected number of sequences in the region i, $S_{i}$ the available number of sequences in the region i, and $C_{i}$ the number of cases in the region i. ∑C is the total number of annual cases in all regions.

For years after 1990, when the number of available sequences was lower than expected value, the first was identified as sample size. When the number of available sequences was greater than the expected value, the expected value was determined as the sample size. In the years prior to 1990, all available sequences were included. Random sampling was then carried out based on the size determined.

#### 2.2.2. Calculate Pairwise Distance for the Sequences

After sampling, the genetic distance between the pairs of sampling sequences was calculated by the PHYLogeny Inference Package (PHYLIP) [40], version 3.697.

#### 2.2.3. Mutation Network Construction Procedure

The construction procedure of the networks is briefly described here. Firstly, the initial network was connected on the basis of a threshold and the threshold for connecting two nodes was the 99th percentile of the minimum genetic distance distribution of all distances between nodes. Secondly, the direction was determined based on the connected network by time. The direction is from the previous time to the following. Finally, to further reduce bias, random sampling and network construction were repeated 100 times at each stage and for the four serotypes individually. The detailed procedure of the method can be checked in the previous study [33].

### 2.3. Measurement of the Circulation Indicators

Four circulation indicators, including local intensity, betweenness centrality, tip frequency and persistence time, were generated on the basis of the network. The local intensity (*Local*) was calculated as the degree of the nodes in the same region and was weighted by the weight of the edges. With respect to the betweenness centrality (*Between*), it was a measurement of how often a node is on the shortest path between two other nodes in the network. It assumes that important nodes are connected to other nodes. The definition of the tip frequency (*Tip*) for a region is the frequency of the tip nodes whose output is 0 based on the directional network, which indicates that the strain can stop circulating and disappear in this region. However, it cannot rule out that samples were not collected after that year. In order to avoid this situation, nodes from the last two years at each stage of the network were not considered top nodes. For each network, the three indicators for each region are calculated as follows in each year.

(2) $${Local}_{i}=\frac{\sum w}{{Seq}_{i}}$$

For a specific year, ${Local}_{i}$ represents the local intensity of the region *i*. ${Seq}_{i}$ represents the total number of sequences in region *i* in the year. ∑w represents the sum of the weights of the edges between the nodes of region *i* in the year.

(3) $$B_{j}=\sum_{s,t\in N}\frac{n_{st\left(j\right)}}{n_{st}}$$

$B_{j}$ represents the betweenness centrality of node *j*. $n_{st}$ represents the shortest path between nodes s and t, and $n_{st(j)}$ represents the shortest path between nodes s and t passing through node j.

(4) $${Between}_{i}=\frac{\sum B}{{Seq}_{i}}$$

For a specific year, ${Between}_{i}$ represents the betweenness centrality of the region *i*. ${Seq}_{i}$ represents the total number of sequences of the region *i* in the year. ∑B represents the sum of betweenness centrality of the nodes of the region *i* in the year.

(5) $${Tip}_{i}=\frac{\sum n}{{Seq}_{i}}$$

For a specific year, ${Tip}_{i}$ represents tip frequency of the region *i*. ${Seq}_{i}$ represents the total number of sequences of the region *i* in the year. ∑n represents the sum of tip nodes of the region *i* in the year.

Furthermore, the three indicators of a region in a given year were finally calculated as the average of 100 random sampling networks. To check the dynamics of the different indicators over three decades from 1990 to 2019, the average of each indictor was calculated in the 1990s, 2000s, and 2010s for each region.

In regard to the persistence time, based on the constructed mutation network, random walks were carried out to find the circulation routes of the dengue virus. First, the initial node was randomly selected based on the PageRank value of each node in the network [33]. The higher the PageRank value of a node, the greater the probability of being selected. Then, the circulation route from the starting node to the end node was obtained based on the weight of the edges. Overall, a total of 10,000 transmission routes for a network were achieved. Combined with the 100 random sampling networks, 1,000,000 circulation routes were ultimately considered at each stage. The routes from the same region during the phase were identified. Then, among these routes, the year between the beginning of the route and the last year of the route was calculated. Since the length of the year for the circulation routes indicates to some extent the persistence time of the dengue virus, the average distribution for the length of the year was obtained as the persistence time of the dengue virus for a given region. Therefore, in this study, the period of persistence time was not less than one year. And if the persistence time in a given region was found, it was considered the dengue persistence in that region. Meanwhile, the change and dynamics of the persistence time in different regions in the 1990s, 2000s, and 2010s were described based on the three stages of the networks.

### 2.4. Classification of Circulation Roles Based on Circulation Indicators

It is of great importance to identify the *source, hub,* and *destination* regions during the circulation of dengue to achieve better and more accurate prevention and control. Consequently, the definition of these three circulation roles was carried out on the basis of the circulation indicators mentioned above. The standard for defining a *source* was that the local intensity for a particular region was greater than the average of the regions. A *hub* should play an intermediate role, so the standard for defining a *hub* was that the betweenness centrality for a given region was higher than the average values for all regions. The *destination* of circulation was considered to be the region in which certain strains die or disappear, so if the tip frequency of a specific region was higher than the mean values, it would be considered as the destination.

### 2.5. Dynamic Change of Three Circulation Roles

To better understand the global circulation dynamics of dengue, changes in spatial distribution and mean latitude were described over the three decades. The number of regions for the three circulation roles was calculated and the detailed spatial distributions were mapped over decades. Cochrane–Mantel–Haenszel (CMH) tests were carried out to test the significant trend of changes. At the same time, Spearman’s correlation analysis was performed to determine whether there is a correlation between the change in the number of regions with circulation roles and sequences over the decades. In addition, the mean latitude between different decades was compared with the Kruskal–Wallis H-test (KWH).

### 2.6. Dynamic Change of Persistence Time

To better understand the dynamic change in the persistence time over decades for dengue, we examined the change in the number of regions with persistence and the difference in the persistence time between the three decades. KWH was used to determine whether there is a statistically significant difference in the duration over decades. In addition, the CMH was conducted to test whether there was a trend. As mentioned above, Spearman correlation analysis was conducted to test whether there is a correlation between the change in the number of regions with sequences and the persistence time.

Furthermore, the persistence time for regions with specific circulation roles was calculated and compared with regions without specific circulation roles at each stage. Wilcoxon rank sum tests were used to test whether there is a statistically significant difference between regions with circulation roles and regions without, only for decades 2000s and 2010s with sufficient data, for the four serotypes separately.

### 2.7. Correlation Analysis

In addition, Spearman’s coefficient was calculated to investigate the correlation between these four indicators and different factors. There were 24 factors that are included and divided into five categories, including climate, forest, socioeconomic, population, and airline. Climate variables include diurnal temperature range, cloud cover, ground frost frequency, potential evapotranspiration, precipitation, mean temperature, maximum temperature, minimum temperature, rain days, and relative humidity. The forest variable was the forest area. And the socioeconomic variables included GDP, physicians per 1000 people, and secondary school enrollment. Population variables included population density, urban population, rural population, and urban population growth. Airline and related variables included internal passenger flow, external passenger flow, case imported by air, cases exported by air, and intra-regional cases by air. The detailed description of these variables is shown in Tables S2–S4. Spearman’s coefficient was first calculated between the factors to check the collinearity between the factors. There was a greater correlation than 0.85 between mean temperature, maximum temperature, minimum temperature and vapor pressure. As a result, the mean temperature remained to represent the temperature condition in a region. Finally, the correlation between the four indicators above and the last 21 factors was examined with Spearman’s coefficient.

### 2.8. Machine Learning Models

As the global circulation of dengue is influenced by several factors, in order to clarify the important determinants for different circulation roles and persistence, three machine learning models have been developed to reduce prejudices caused by a single method. Separate models were constructed to distinguish whether a region is a source or not, whether it is a hub or not, and whether it is a destination or not. Meanwhile, the model distinguishes whether the persistence of dengue virus is in a region or not. Random forest, eXtreme Gradient Boosting (XGBoost), and Light Gradient Boosting Machine (LGBM) were compared. The implementation of the three classification models was conducted using Python 3.9 using the sklearn package. Specifically, the maximum depth of the random forest model was configured to 2, and all other hyperparameters were assigned their default values. Similarly, the hyperparameters of the XGBoost and LGBM models were maintained with their default settings. The train set and the test set were divided based on the 7:3 ratio and the test set was used to test the performance of the models. The evaluation metrics used include accuracy, recall, and F1-score values. Synthetic Minority Over-Sampling Technique (SMOTE) was applied to the training set to reduce the influence of imbalanced samples. The SHAP framework was used to obtain the contributions of different factors [34,35]. Given the completeness of the data, the data from 2011 to 2019 were used and factors from correlation analysis were input into the models.

## 3. Results

### 3.1. Global Distribution for Different Circulation Roles and Persistence

To investigate the global circulation dynamics of dengue over three decades from 1990 to 2019, four circulation indicators were proposed. Firstly, the local intensity determines the degree of the virus circulation within the same region, a measure of endemicity to some extent. Secondly, the betweenness centrality indicates the importance for a region as the role of a hub. The higher the value of betweenness centrality a region is, the more important it is for the transmission of dengue. Thirdly, the tip frequency indicates the probability that strains stop circulating and disappear in a region. Finally, the persistence time is calculated as the duration of dengue virus continuous circulation in a given region and persistence is defined as persistence time no less than one year (see Section 2). These circulation indicators reflect different aspects of the global circulation of the dengue virus. The average normalized value of local intensity, betweenness centrality and tip frequency in 1990s, 2000s, and 2010s for each region is shown in Figures S1–S4.

**Figure 1:**
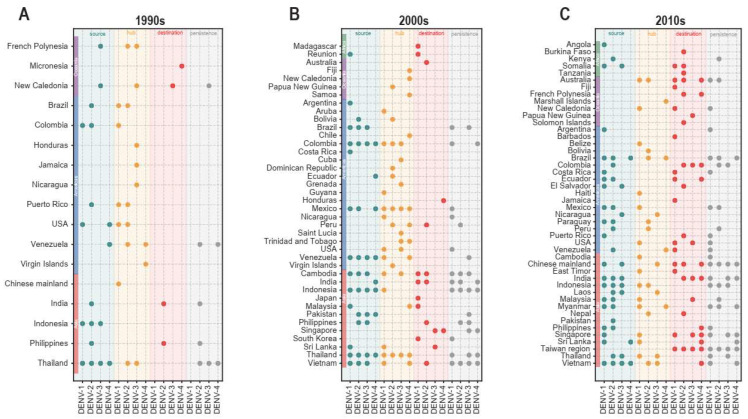
Circulation roles and persistence in three decades. (**A**–**C**) show the results of decades of 1990s, 2000s, and 2010s, respectively. The horizontal axis is different serotypes, and the vertical axis is the regions that are classified as the roles. Source, hub, destination, and persistence are colored by teal, orange, red, and grey, respectively. The points in the figure represent the regions were classified as the roles or with persistence time found there. The regions in the red, blue, purple, and green rectangles of vertical axis represent Asia, Americas, Oceania, and Africa, respectively.

Furthermore, based on the indicator that the value of the local intensity is higher than its average value, the regions were classified as sources. Similarly, based on the indicators of the value of betweenness centrality and tip frequency are greater than their average values, regions were classified as hubs and destinations, respectively. With the exception of regions as destinations for DENV-1 and regions with persistence that were not observed in 1990s, regions with circulation roles and with persistence for all four dengue serotypes over the three decades are shown in Figure 1. The number of regions with different circulation roles in different decades can be checked in Table S5. Figure 1 shows that Thailand was the source of all four serotypes in the 1990s and 2000s. In addition, Venezuela, Colombia, and Indonesia were the source of all four serotypes in the 2000s, while Vietnam was the source of all four serotypes in 2010s. In addition, Thailand and Mexico are the hubs of all four serotypes in the 2000s. Taiwan region, is the destination of the four serotypes in 2010s. In addition, it is interesting to note that the destinations are located mainly in island regions, such as Madagascar, Fiji, Jamaica, Barbados for DENV-1, French Polynesia and Solomon Islands for DENV-2, New Caledonia, Sri Lanka, Papua New Guinea for DENV-3, and Micronesia, Honduras, French Polynesia for DENV-4.

Furthermore, as illustrated in Figure 1, persistence was observed in most regions with circulation roles. Nonetheless, persistence was also identified in regions without circulation roles (see Figure S5 for more details). Given the limited number of regions that showed dengue persistence in the 1990s, the median persistence time for regions with circulation roles during the 2000s and 2010s is presented in Table 1. Wilcoxon rank sum tests were employed to determine whether the persistence times are statistically different between regions with and without circulation roles. It was discovered that the persistence time for DENV-1 hubs in the 2000s is significantly longer than that for non-role regions. Additionally, the persistence time for DENV-2 destinations in the 2000s is significantly longer than that for non-role regions, and the same applies to DENV-3 sources. Furthermore, the persistence times for regions with circulation roles of DENV-1 and sources of DENV-2 in the 2010s are statistically significantly longer than those for non-role regions. However, in the case of DENV-4, no statistically significant results were found, possibly due to a much lower number of available sequences. Detailed results are shown in Table 1.

### 3.2. Spatial and Temporal Dynamics for Different Circulation Roles and Persistence

**Figure 2:**
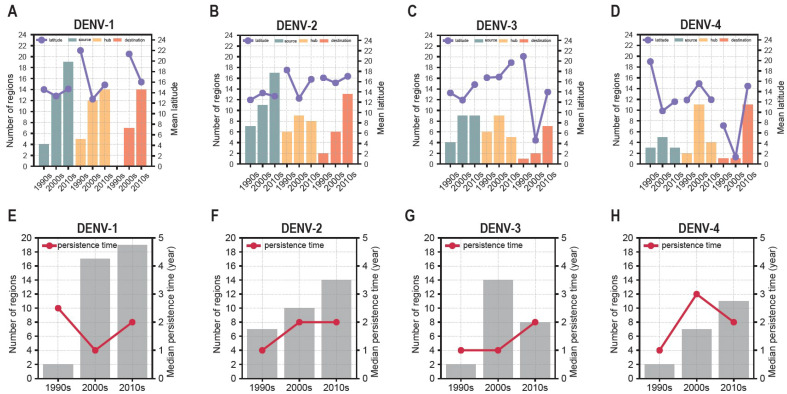
Change of mean latitude of roles and median persistence time over the three decades. (**A**–**D**) show the mean latitude change of DENV-1, 2, 3, and 4, respectively. The horizontal axis is the three decades. The left vertical axis represents number of regions, and the right one represents mean latitude. The teal, orange, and red bars represent number of source, hub, and destination, respectively. The solid medium purple line represents mean latitude. (**E**–**H**) show the median persistence time change of DENV-1, 2, 3, and 4, respectively. The horizontal axis is the three decades. The left vertical axis represents number of regions, and the right one represents median persistence time. The grey bars represent number of regions with dengue persistence. The solid crimson line represents median persistence time.

To gain a more comprehensive understanding of the spatial and temporal variations of the three circulation roles and their persistence over the three decades, their dynamic changes have been studied. Firstly, the spatial distributions of the regions with circulation roles and the changes in their mean latitude were assessed (see Figure S6). The number of regions with circulation roles and their mean latitudes over the three decades are presented in Figure 2A–D.

#### 3.2.1. DENV-1

As illustrated in Figure S6A, the sources of DENV-1 were distributed in both the Americas and Asia, with a notable concentration in Southeast Asia during the 1990s. With the arrival of the 2000s, there was an increase in the number of sources. By the 2010s, the sources were dispersed on a broader geographical scale, extending to Africa. Regarding the hubs for DENV-1, these were initially located at higher latitudes in the 1990s, such as the Chinese mainland and the USA. Subsequently, regions at lower latitudes also emerged as hubs, including Indonesia and Vietnam. By the 2010s, the hubs for DENV-1 were established across the Americas, Asia, and Oceania. Concerning the destinations of DENV-1, no destinations were observed in the 1990s, but over the subsequent two decades, there was a gradual increase, with destinations identified in numerous island regions. The number of DENV-1 circulation roles increased significantly as evidenced by the CMH test (*p* < 0.05), and correlation analysis suggests that this upward trend is not significantly linked to changes in the number of regions with sequences (*p* > 0.05). Detailed results are presented in Figure 2A and Table S5.

#### 3.2.2. DENV-2

Regarding DENV-2, the main sources were predominantly located in Asia and the Americas. Over the course of three decades, the quantity of sources remained largely unchanged according to the CMH test (*p* > 0.05). Concerning the hubs, the majority were situated in the Americas during the first years, with additional hubs emerging in Asia and Oceania over the succeeding two decades. Moreover, the destinations for DENV-2 were initially situated in Asia, subsequently extending to Oceania, America, and even Africa by the 2010s. The quantity of destinations exhibited a gradual increase throughout the three decades as evidenced by the CMH test (*p* < 0.05), and this upward trend was not significantly correlated with the number of regions with sequences, based on the correlation analysis (*p* > 0.05). For more information on the spatial distribution, see Figure S6B and Figure 2B and Table S5, which provide detailed numerical data.

#### 3.2.3. DENV-3

The circulation dynamics of DENV-3 reveal that the sources were mainly concentrated in Southeast Asia during the 1990s, with subsequent expansion in South Asia and the Americas over the following two decades. During the 1990s, hubs for DENV-3 were located in both Asia and the Americas. In the 2000s, these hubs were primarily situated in the Americas, while in the 2010s, the majority were found in Asia. Concerning the number of destinations, there were only a few in the 1990s and 2000s, but this number increased significantly in the 2010s (*p* < 0.05). The correlation analysis indicates that this upward trend is not significantly associated with the number of regions with sequences (*p* > 0.05). It was observed that a number of destinations were distributed across Asia and the Americas in the 2010s. Detailed spatial distribution of the regions with the three circulation roles can be examined in Figure S6C, and detailed numerical data can be found in Figure 2C and Table S5.

#### 3.2.4. DENV-4

With regard to the circulation roles of DENV-4, its spatial coverage was notably less extensive in comparison to the other three serotypes (Figure 2D and Table S5). Specifically, in terms of the source regions, there were approximately three to five per decade. These sources were predominantly located in Southeast Asia and the Americas, such as Thailand and Venezuela during the 1990s and 2000s, as well as Brazil and Vietnam in the 2010s. As for the hubs, they were primarily located in the Americas during the 1990s, and they expanded to include both Southeast Asia and the Americas in the subsequent two decades. The number of destinations was lower in the initial two decades, but by the 2010s, they were mainly situated in Asia, the Americas, and Oceania. The number of destinations has increased statistically significantly over the decades (*p* < 0.05); however, correlation analysis suggests that this increasing trend may be influenced by the variation in the number of regions with sequences (*p* < 0.05). Figure S6D illustrates the spatial distribution details, while changes in the numbers can be observed in Figure 2D and Table S5.

#### 3.2.5. Persistence Time

Moreover, to investigate the dynamic pattern of persistence time, the median persistence times over the three decades are elaborated upon (Figure S5). It is therefore important to highlight that persistence (defined as persistence time equal to or greater than one year, as detailed in the Section 2) for all four serotypes was documented in 12 countries or regions, namely Thailand, Brazil, Indonesia, India, Myanmar, Singapore, Vietnam, the Philippines, Venezuela, Colombia, Chinese mainland, and Taiwan region. Additionally, the maximum median persistence time for DENV-1 was recorded as 6 years in Mexico, Brazil, and Argentina during the 2010s. The longest median persistence time for DENV-2 was identified as 7 years in Mexico and Brazil during the 2010s. The maximum median persistence time for DENV-3 was 4 years in the Philippines during the 2010s, while for DENV-4, it was 5 years in Singapore and Colombia during the 2000s. Generally, as illustrated in Figure 2E–H, the persistence of dengue was evident in an increasing number of countries or regions. According to the CMH test, a significant trend of increasing for DENV-1 was observed, and this trend did not show a significant correlation to the number of regions with sequences (*p* > 0.05). Regarding DENV-3 and DENV-4, a significant increase trend was also detected (*p* < 0.05), although correlation analysis suggests that this trend may be influenced by changes in the number of regions with sequences (*p* < 0.05). Conversely, no significant increase trend for DENV-2 was noted (*p* > 0.05), based on the CMH test.

In summary, the spatial distributions of regions with dengue circulation roles have evolved over the decades. According to the CMH tests, the number of DENV-1, 2, and 3 destinations increased significantly over the three decades (*p* < 0.05). Concurrently, the number of sources and hubs for DENV-1 increased during the same period (*p* < 0.05). Contrarily, this trend was not observed in the other three serotypes. The detailed results are presented in Table S5. Furthermore, there is no statistically significant difference in the mean latitude of these regions across different decades for any circulation role across the four serotypes (*p* > 0.05). Consequently, the key regions with circulation roles for dengue remain predominantly situated within the latitude of 10–20°, exhibiting no significant change over the decades (Figure 2A–D). Additionally, the number of regions with DENV-1 persistence is increasing considerably. However, the median persistence time is approximately 1 to 3 years for the four serotypes, with no statistically significant variation observed across decades (*p* > 0.05) (Figure 2E–H).

### 3.3. Determinants for Different Circulation Roles and Persistence

Moreover, the identification of factors associated with various circulation roles and dengue persistence is beneficial for the development of targeted and effective prevention and control strategies. Consequently, Spearman’s correlation coefficient was initially employed to quantify the association between circulation indicators and a range of factors. As illustrated in Figure S7, it was determined that climate, population, socioeconomic, and forest factors hold significant correlations with the dengue circulation indicators to varying degrees.

**Figure 3:**
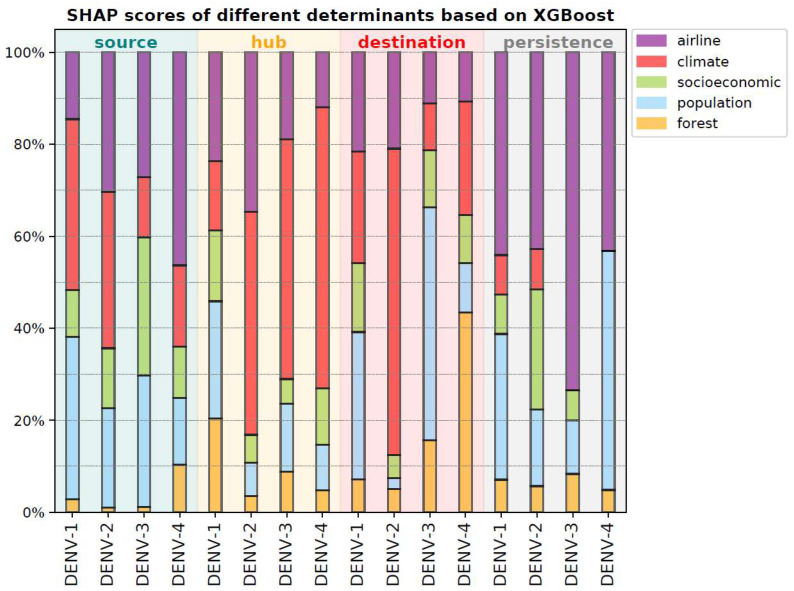
SHAP scores of different determinants based on XGBoost. The horizontal axis is the four serotypes, and the vertical axis is the proportion of SHAP scores of different determinants. Five categories of the determinants, including climate, airline, population, socioeconomic, and forest factors, are colored as showed in the right legend. Results for the source, hub, destination, and persistence are colored by teal, orange, red, and grey, respectively.

Nevertheless, the global circulation of dengue is subject to many factors and their complex interactions. Consequently, traditional correlation analyses, which are predominantly linear, may have limited effectiveness. To address this, significant determinants related to different circulation roles and persistence were delineated using machine learning models, which are more adept at handling nonlinear relationships. In this study, three prominent machine learning models were employed. Among these, the LGBM model performed inadequately in terms of persistence time, prompting the selection of XGBoost and Random Forest for further analysis. The performance metrics of these models are presented in Table S6. Additionally, SHAP values for each determinant were evaluated, and the SHAP score for each determinant category was computed as the proportion of the aggregate absolute SHAP values for each determinant within each category. The SHAP scores for the various determinant categories are summarized and illustrated (Figure 3, Figures S8 and S9). The detailed results for the XGBoost model are subsequently presented.

Precisely, the XGBoost model demonstrates the accuracy of classifying the source of DENV-1, DENV-2, DENV-3, and DENV-4 at 80%, 100%, 82%, and 88%, respectively. The comprehensive SHAP values for the factors are presented in Figures S10–S13, while the SHAP scores for different determinant categories are illustrated in Figure 3. From the highest proportions of SHAP scores, climate emerges as the most critical determinant for the sources of DENV-1 and DENV-2, with 37% and 34%, respectively. The comprehensive SHAP score values are available in Table S7. Furthermore, socioeconomic and airline factors are pivotal determinants for the sources of DENV-3 and DENV-4, accounting for 30% and 46%, respectively. Subsequently, the accuracy of the model in classifying the hubs for DENV-1, DENV-2, DENV-3, and DENV-4 is 83%, 100%, 88%, and 94%, respectively (see Figures S14–S17 for detailed SHAP values). Climate is identified as an essential determinant for DENV-2, DENV-3, and DENV-4 hubs, accounting for 49%, 52%, and 61%, respectively, according to the largest SHAP score proportions (Table S7). Additionally, population accounts for the largest share (25%) of DENV-1 hub determinants, followed by the airline (24%). Moreover, the accuracy of model for classifying destinations for DENV-1, DENV-2, DENV-3, and DENV-4 is 97%, 86%, 88%, and 94%, respectively (see Figures S18–S21 for detailed SHAP values). Population is the key determinant of the DENV-1 and DENV-3 destinations, occupying 32% and 51%, respectively, based on the largest SHAP score proportions (Table S7). Additionally, climate (67%) and forest (43%) are significant determinants for DENV-2 and DENV-4 destinations, respectively. Furthermore, the accuracy of the XGBoost model for the persistence of DENV-1, DENV-2, DENV-3, and DENV-4 is 67%, 62%, 71%, and 50%, respectively (see Figures S22–S25 for SHAP value details). From the predominant SHAP score proportions, airline is the principal determinant for the persistence of DENV-1, DENV-2, and DENV-3, accounting for 44%, 43%, and 73%, respectively (Table S7). Additionally, population (52%) is the leading determinant for the persistence of DENV-4, followed by airline (43%).

In summary, as illustrated in Figure 3, the five categories of factors—forest, population, socioeconomic, climate, and airline—serve as significant determinants in relation to the roles of circulation and the persistence of dengue. Moreover, climate frequently emerges as a crucial determinant in the role of circulation, whereas airline factors are pivotal to the persistence of dengue. Consistent results can be observed by analysis with the Random Forest and LGBM models (Figures S8 and S9).

## 4. Discussions

This study comprehensively and systematically investigates the dynamics of global circulation of the four serotypes of the dengue virus utilizing genetic data. By employing a network-based method, the characteristics of global dengue transmission, as delineated by three circulation roles and persistence time, have been studied over the three decades from 1990 to 2019. The findings indicate that Thailand, Indonesia, and Vietnam in Asia, as well as Venezuela and Colombia in the Americas, have historically served as sources for all four serotypes in different decades, while destinations were identified in numerous island regions. Over the decades, there was a significant increase in the number of circulation roles and regions with persistence time for DENV-1. However, there was no observed difference in the median persistence time for the four serotypes, nor in the mean latitude for regions with circulation roles. Additionally, factors such as forest cover, population, socioeconomic conditions, climate, and airline were all significant determinants of circulation roles and dengue persistence. Climate frequently emerged as a critical determinant for circulation roles, whereas airline was crucial for persistence. The findings of this study are key to facilitating targeted dengue prevention and control strategies.

To comprehensively investigate the global dynamics of dengue circulation, this study evaluates four indicators that reflect varying circulation statuses: persistence time, local intensity, betweenness centrality, and tip frequency. Notably, persistence time has been systematically quantified as a measure of the duration of continuous circulation for a specific lineage of the dengue virus. A longer persistence time in a particular region correlates with a higher likelihood of that region being a source of the dengue virus, thereby providing valuable insight for resource allocation, the implementation of precise countermeasures, and targeted dengue control. The study observed that the median persistence time of DENV-1 in Brazil was six years during the period from 2010 to 2019. This finding aligns with the results of another study, which indicated that the outbreaks in 2018–2019 were attributable to local DENV-1 lineages that persisted for five to ten years in Brazil [21]. Consequently, regions identified with prolonged dengue virus persistence in the study warrant additional attention and increased healthcare resources for improved dengue control. Additionally, the study examines changes in both the number of regions and median persistence time over three decades. While the median persistence time is approximately between one to three years, no statistically significant differences were detected over time. Nonetheless, the increase in the number of regions exhibiting dengue persistence over time is alarming and warrants further investigation.

Furthermore, three circulation roles are identified, namely source, hub, and destination, which are instrumental in managing dengue. Since the spread of the dengue virus varies over time, these three roles were identified over three decades. It was observed that Thailand served as the source for all four serotypes in the early decade, aligning with its function in Asia [19]. However, it could also function as a hub during certain decades as the dengue virus extended to a broader spatial dimension. Thailand may assume multiple roles in the global circulation of dengue due to a variety of factors. Similar phenomena were found in other regions, including Indonesia, Vietnam, Brazil, Venezuela, Colombia, and Mexico. This pattern may result from heightened dengue activity in these regions coupled with substantial population mobility and traffic, resulting in the spread of the dengue virus as a complex network. In this context, certain regions may manifest the characteristics of one or more circulation roles throughout the decade rather than a single role. This situation required the implementation of targeted strategies to control dengue in these areas, which would also be beneficial globally. Additionally, it was identified that Thailand, Indonesia, and Vietnam were the sources of all four serotypes, as well as Venezuela and Colombia in the Americas. Notably, the three primary circulation roles were present in both Asia and the Americas over the past three decades. Considering a prior study indicating that inter-continental circulation is rare [41], it can be suggested that Asia and the Americas could constitute the two dengue epicenters globally. Furthermore, the main sources found in this study have been reported in many existing studies. For example, a majority of the sources of DENV-1 in this study have been reported in the existing literature or reports [22,42,43,44]. However, as many existing studies focus on the limited region, there may be sources that have not been found. From a global point of view, unknown sources could also be observed. Although the roles of source and hub have been previously highlighted, role of the destination has received limited attention. Identifying destinations is crucial as they represent regions where viruses are eradicated. Destinations were located in several island regions, such as Madagascar, Fiji, Jamaica, and Barbados for DENV-1; French Polynesia and the Solomon Islands for DENV-2; New Caledonia, Sri Lanka, Papua New Guinea for DENV-3; and Micronesia and French Polynesia for DENV-4, potentially attributable to the geographical isolation impeding the spread of the dengue virus.

The study focused on dynamic changes in the spatial distribution and mean latitude of the three circulation roles. It was observed that there was an increasing trend in the number of circulation roles, and spatial distribution evolved over the three decades. Despite changes in the mean latitude of the three roles over time, no significant difference was detected, with the mean latitude remaining approximately between 10–20°. Consequently, the dengue virus actively circulates within latitude regions between 10–20° worldwide, exhibiting no notable changes over the decades. This corresponds to previous knowledge that dengue occurs predominantly in tropical and subtropical areas around the world [1]. Although dengue is expanding to higher latitudes [45], its activity is not as pronounced as in lower latitudes. However, a gradual spread of dengue to higher altitudes can be observed, possibly due to the spread of mosquito vectors to higher altitudes, especially in the regions at high altitudes in Europe and America. Therefore, urgent measures are necessary to prevent regions at higher latitudes from becoming dengue-active areas. Furthermore, when comparing the persistence time between the key roles and non-roles, it was found that the persistence time of key roles surpasses that of non-roles of DENV-1, DENV-2, and DENV-3. In other words, these regions not only play a key role in the global circulation of dengue but also serve as sources of the virus. Hence, it is prudent to allocate more efforts and resources to these regions for more effective dengue control.

In this study, it was demonstrated that the global spread of the dengue virus is affected by systematic factors. Specifically, the study identified five categories of influential factors: forest, population, socioeconomic, climate, and airline factors, as critical determinants of the roles in the circulation and persistence of dengue. Overall, it was determined that climate factors generally serve as the primary determinants of circulation roles, while airline factors are crucial to the persistence of dengue. Firstly, climate factors in this study include the diurnal temperature range, cloud cover, ground frost frequency, potential evapotranspiration, precipitation, mean temperature, rain days, and relative humidity. Notably, some of these factors, such as temperature and precipitation, have been extensively investigated in previous research. Indeed, the impact of temperature and precipitation on dengue transmission has been substantiated [46]. For instance, research conducted in Argentina has evidenced the significant influence of temperature on the transmission of dengue virus [47]. Moreover, climate change can significantly impact long-term dengue virus circulation by altering temperature and precipitation patterns, which affect mosquito breeding and virus replication. Warmer temperatures can expand the geographic range of Aedes mosquitoes into previously cooler regions and lengthen the transmission season [48]. Changing precipitation can create more breeding sites or force water storage in drought-prone areas, both increasing mosquito habitats [49]. These shifts may lead to higher transmission intensity in endemic areas and introduce dengue to new regions. As a result, regional risk profiles will evolve, requiring adaptive surveillance and climate-informed public health strategies. Additionally, the relationship between dengue circulation and factors such as cloud cover and potential evapotranspiration is still insufficiently understood. The analysis of SHAP values revealed that potential evapotranspiration and cloud cover are paramount determinants of dengue circulation roles within this study, warranting further investigation to elucidate the mechanisms involved. Furthermore, airline factors include external and internal air passenger flow, cases exported by air, cases imported by air, and intra-regional cases by air. It was notably observed that airline factors are pivotal determinants of dengue’s persistence. Specifically, intra-regional cases by air consistently emerged as the main airline factor based on SHAP values, indicating the significance of domestic air travel in sustaining dengue persistence. Besides climate factors, it was observed that airline factors significantly influence the circulation of dengue, particularly in the context of cases imported and exported by air, as derived from SHAP values. Coupled with findings indicating that destinations were predominantly island regions, it can be inferred that international air travel is a significant driver of global dengue circulation. In fact, another study indicated that increased airline travel facilitates the spread of dengue in Asia [19], consistent with the findings regarding the global propagation of dengue observed in this study. Consequently, reinforcing entry and exit quarantine measures may constitute a critical approach to mitigating the global spread of the dengue virus. The pivotal factors identified by this study could serve as valuable references for dengue prevention and control. Furthermore, the expansion of mosquito vectors and dengue into new areas, particularly in Europe [50], the Americas, and high-altitude regions such as Nepal, and the possible drivers, including climate change, urbanization and human travel, should be considered to control dengue. Climate change enables Aedes mosquitoes to survive at higher altitudes by creating warmer conditions that support breeding and viral transmission [48,49]. Urbanization and poor water storage in high-altitude areas provide ideal breeding sites, as uncovered containers and construction debris collect stagnant water [51]. Human travel and trade introduce the virus to new regions, while transported goods may carry mosquito eggs, spreading dengue to non-endemic zones [50].

In the current study, it is observed that both the homogeneous pattern of the four serotypes and the heterogeneity among them are evident. Several factors may contribute to the heterogeneity between the serotypes. For example, the global distribution of different serotypes varies, with DENV-1 potentially covering a larger geographical area, as evidenced by increased reports in recent years [12]. This trend may result in the finding that the number of circulation roles and regions with persistent DENV-1 has increased significantly over the decades. Additionally, the heterogeneity of antigenicity and pathogenicity among the serotypes may contribute to this observation. It has been noted that serotypes exhibit a trend of increasing antigenic distance from previous strains, followed by a gradual convergence to more similar strains. However, these fluctuations in antigenic distance were found to be significant solely for DENV-1 and DENV-2 in a previous study [52]. Furthermore, DENV serotype significantly influences the clinical manifestations and severity of dengue, with DENV-2 infections more frequently linked to the need for fluid expansion, shock, and prolonged hospital stays [53]. Consequently, there may be fundamental differences between the four serotypes contributing to the observed heterogeneity in this study, which requires further research to elucidate these differences to improve dengue control.

However, the study has certain limitations. Firstly, the insufficient genomic surveillance in various countries or regions results in a lack of available sequences, particularly a notable paucity of data in many regions of Africa, which leads to an underestimation of their contribution to dengue circulation. Owing to data constraints, serotype rather than genotype was utilized for analysis within the study. Moreover, to minimize the bias of genetic data in each region, in this study, genetic data sampling corrected for the number of cases in each region was used to construct the global transmission networks. This is a possible reduction to a certain extent of the effect of limited data in some regions. If more sequence data are to be collected in these areas in the future, their role in the global spread of dengue can be identified more precisely. Secondly, the absence of serotype-specific case data for each country or region covered in this study prevents the individual consideration of dengue cases for each serotype. The inclusion of serotype-specific case data in further study would indeed improve the precision of the analyses and provide a better understanding of the circulation patterns of the serotypes. Moreover, mild or asymptomatic cases were not included due to the unavailability of data. Thirdly, the factors incorporated in this study might not be sufficiently comprehensive, including aspects such as vector density, large-scale institutional interventions, and the effects of heterogeneity in weather conditions. More factors should be considered in the future. Lastly, the study employs dichotomous models, which are incapable of discerning differences within the same category. Future research, utilizing a larger sample size, may better elucidate the differences between regions. Nonetheless, the factors identified in this study can serve as a reference for subsequent research and dengue control practices.

In light of the global expansion of dengue and its sporadic outbreaks over several decades, this study contributes to a more comprehensive understanding of the global circulation of dengue, helping to mitigating its threat to human populations.

## Figures and Tables

**Table 1:** Median persistence time of different roles in two decades.

Serotype	Decade	Median (Interquartile Range)			
		Source	Hub	Destination	Non-Role
DENV-1	2000s	1 (0, 1.5)	1 * (0, 1.25)	0 (0, 1)	0 (0, 0)
	2010s	1 * (0, 2)	1 * (0, 2)	1 * (0, 1.75)	0 (0, 0)
DENV-2	2000s	0 (0, 1.5)	0 (0, 0)	2 * (2, 2.75)	0 (0, 0)
	2010s	1 * (0, 3)	1 (0.75, 2.5)	0 (0, 2)	0 (0, 0)
DENV-3	2000s	1 * (1, 2)	0 (0, 0)	0.5 (0.25, 0.75)	0 (0, 0)
	2010s	0 (0, 2)	0 (0, 2)	1 (0, 1.5)	0 (0, 0)
DENV-4	2000s	1 (0, 2)	0 (0, 0)	5 (5, 5)	0 (0, 0)
	2010s	3 (2, 3.5)	0.5 (0, 1.25)	1 (0, 2.5)	0 (0, 1)

* denotes statistically significant compared to non-role (*p* < 0.05).

## Data Availability

The data presented in this study were derived from the following resources available in the public domain: [WHO dengue data application (https://ntdhq.shinyapps.io/dengue5/, accessed on 26 July 2025), Official Aviation Guide (https://analytics.oag.com/analyser-client/home, accessed on 26 July 2025), CRU CY4.05 dataset (https://data.ceda.ac.uk/badc/cru/data/cru_cy/cru_cy_4.05/data, accessed on 26 July 2025), World Bank (https://databank.worldbank.org/reports.aspx?source=World-Development-Indicators, accessed on 26 July 2025) and Dengue virus database (https://www.ncbi.nlm.nih.gov/genomes/VirusVariation/Database/nph-select.cgi, accessed on 26 July 2025)].

## Supplementary Materials

The following supporting information can be downloaded at: https://www.mdpi.com/article/10.3390/v17081078/s1. Table S1: Number of regions with sequences for four serotypes of dengue virus in three decades. Table S2: Description of socioeconomic, population, and forest factors. Table S3: Description of climate factors. Table S4: Description of airline factors. Table S5: Change of the number of roles and the mean number in the three decades. Table S6: Performances of three machine learning models. Table S7: SHAP score values of different determinants based on XGBoost. Figure S1: Three circulation indicators in three decades of DENV-1. Figure S2: Three circulation indicators in three decades of DENV-2. Figure S3: Three circulation indicators in three decades of DENV-3. Figure S4: Three circulation indicators in three decades of DENV-4. Figure S5: Median persistence time in different regions for three decades. Figure S6: Spatial distribution of different circulation roles during 1990–2019. Figure S7: Spearman’s coefficient between the four indicators and factors. Figure S8: SHAP scores of different determinants based on Random Forest. Figure S9: SHAP scores of different determinants based on LGBM. Figure S10: SHAP values of features to classify source of DENV-1. Figure S11: SHAP values of features to classify source of DENV-2. Figure S12: SHAP values of features to classify source of DENV-3. Figure S13: SHAP values of features to classify source of DENV-4. Figure S14: SHAP values of features to classify hub of DENV-1. Figure S15: SHAP values of features to classify hub of DENV-2. Figure S16: SHAP values of features to classify hub of DENV-3. Figure S17: SHAP values of features to classify hub of DENV-4. Figure S18: SHAP values of features to classify destination of DENV-1. Figure S19: SHAP values of features to classify destination of DENV-2. Figure S20: SHAP values of features to classify destination of DENV-3. Figure S21: SHAP values of features to classify destination of DENV-4. Figure S22: SHAP values of features to determine persistence time of DENV-1. Figure S23: SHAP values of features to determine persistence time of DENV-2. Figure S24: SHAP values of features to determine persistence time of DENV-3. Figure S25: SHAP values of features to determine persistence time of DENV-4.
