# Supplementary material for: Global Circulation Dynamics and Its Determinants of Dengue Virus: A Network Evolution and Model Study from 1990 to 2019
Source: Viruses. 2025 Aug 4;17(8):1078. doi: 10.3390/v17081078 (PMC12390658; doi:10.3390/v17081078)
Supplement: Supplementary file 1 [file viruses-17-01078-s001.zip › viruses-3735155-supplementary.pdf]

Supplementary information for

Global circulation dynamics and its determinants of dengue virus: a network evolution and model study from 1990 to 2019

Table S1. Number of regions with sequences for four serotypes of dengue virus in three decades

Serotype	Decade	Number of regions with sequences
DENV-1	1990-1999	35
	2000-2009	171
	2010-2019	166
DENV-2	1990-1999	62
	2000-2009	174
	2010-2019	152
DENV-3	1990-1999	38
	2000-2009	152
	2010-2019	98
DENV-4	1990-1999	19
	2000-2009	86
	2010-2019	87

Table S2. Description of socioeconomic, population and forest factors

Factor	Definition	Units
GDP	GDP per capita is gross domestic product divided by midyear population.	per capita (current US$)
physicians	Physicians include generalist and specialist medical practitioners.	per 1,000 people
secondary school enrollment	Gross enrollment ratio is the ratio of total enrollment, regardless of age, to the population of the age group that officially corresponds to the level of education shown. Secondary education completes the provision of basic education that began at the primary level, and aims at laying the foundations for lifelong learning and human development, by offering more subject- or skill-oriented instruction using more specialized teachers.	% gross
population density	Population density is midyear population divided by land area in square kilometers. Population is based on the de facto definition of population, which counts all residents regardless of legal status or citizenship--except for refugees not permanently settled in the country of asylum, who are generally considered part of the population of their country of source. Land area is a country's total area, excluding area under inland water bodies, national claims to continental shelf, and exclusive economic zones. In most cases the definition of inland water bodies includes major rivers and lakes.	people per sq. km of land area
urban population	Urban population refers to people living in urban areas as defined by national statistical offices. It is calculated using World Bank population estimates and urban ratios from the United Nations World Urbanization Prospects.	-
rural population	Rural population refers to people living in rural areas as defined by national statistical offices. It is calculated as the difference between total population and urban population.	-

urban population growth	Urban population refers to people living in urban areas as defined by national statistical offices. It is calculated using World Bank population estimates and urban ratios from the United Nations World Urbanization Prospects.	annual %
forest area	Forest area is land under natural or planted stands of trees of at least 5 meters in situ, whether productive or not, and excludes tree stands in agricultural production systems (for example, in fruit plantations and agroforestry systems) and trees in urban parks and gardens.	% of land area

Table S3. Description of climate factors

**Factors**	**Units**
Cloud Cover	percentage
Diurnal Temperature Range	degrees Celsius
Ground Frost Frequency	days
Potential Evapotranspiration	mm/day
Precipitation	mm/month
Minimum Temperature	degrees Celsius
Mean Temperature	degrees Celsius
Maximum Temperature	degrees Celsius
Vapour Pressure	hPa
Rain Days	days
Relative humidity	%rh

Table S4. Description of airline factors

**Factor**	**Definition**
External air passenger flow	Number of external air passengers, is equal to the average of number of outbound and inbound air passengers
Internal air passenger flow	Number of internal air passengers
Export flow	Number of outbound air passengers
Import flow	Number of inbound air passengers
Cases exported by air	Number of outbound air passengers × incidence rate of the place of departure
Cases imported by air	Number of inbound air passengers × incidence rate of the place of departure
Intra-regional cases by air	Number of internal air passengers × local incidence rate

Table S5. Change of the number of roles and the mean number in the three decades

**Serotype**	**Decade**	**source**	**hub**	**destination**
**DENV-1**	1990-1999	4	5	0
	2000-2009	13	12	7
	2010-2019	19	14	14
	Mean number	12	10.33	7
	p	0.0001*	0.0244*	0.0000*
**DENV-2**	1990-1999	7	6	2
	2000-2009	11	9	6
	2010-2019	17	8	13

	Mean number	11.67	7.67	7
	p	0.0595	0.4588	0.0031*
**DENV-3**	1990-1999	4	6	1
	2000-2009	9	9	2
	2010-2019	9	5	7
	Mean number	7.33	6.67	3.33
	p	0.1082	0.7675	0.0344*
**DENV-4**	1990-1999	3	2	1
	2000-2009	5	11	1
	2010-2019	3	4	11
	Mean number	3.67	5.67	4.33
	p	0.6877	0.0624	0.0018*

*denotes p <0.05 of the CMH test between the number of roles and the mean number.

Table S6. Performances of three machine learning models

Serotype	Role	Model	Accuracy	Recall	F1
DENV-1	source	xgboost	0.8	0.727272727	0.842105263
		random forest	0.733333333	0.681818182	0.789473684
		LGBM	0.8	0.772727273	0.85
	hub	xgboost	0.833333333	0.636363636	0.736842105
		random forest	0.733333333	0.363636364	0.5
		LGBM	0.766666667	0.545454545	0.631578947
	destination	xgboost	0.966666667	0.8	0.888888889
		random forest	0.9	0.8	0.727272727
		LGBM	0.933333333	0.8	0.8
	Persistence time	xgboost	0.666666667	0.333333333	0.5
		random forest	0.583333333	0.333333333	0.444444444
DENV-2	source	xgboost	1	1	1
		random forest	0.965517241	0.9375	0.967741935
		LGBM	1	1	1
	hub	xgboost	1	1	1
		random forest	1	1	1
		LGBM	1	1	1
	destination	xgboost	0.862068966	0.727272727	0.8
		random forest	0.862068966	0.636363636	0.777777778
		LGBM	0.931034483	0.909090909	0.909090909
	Persistence time	xgboost	0.615384615	0.75	0.545454545
		random forest	0.769230769	0.75	0.666666667
DENV-3	source	xgboost	0.823529412	0.888888889	0.842105263
		random forest	0.823529412	0.888888889	0.842105263
		LGBM	0.764705882	0.777777778	0.777777778
	hub	xgboost	0.882352941	1	0.8
		random forest	0.882352941	1	0.8

		LGBM	0.823529412	1	0.727272727
	destination	xgboost	0.882352941	0.5	0.666666667
		random forest	0.882352941	0.5	0.666666667
		LGBM	0.941176471	0.75	0.857142857
	Persistence time	xgboost	0.714285714	1	0.5
		random forest	0.714285714	1	0.5
DENV-4	source	xgboost	0.882352941	0.5	0.666666667
		random forest	0.882352941	0.5	0.666666667
		LGBM	0.941176471	0.75	0.857142857
	hub	xgboost	0.941176471	1	0.857142857
		random forest	0.941176471	1	0.857142857
		LGBM	0.941176471	1	0.857142857
	destination	xgboost	0.941176471	1	0.941176471
		random forest	1	1	1
		LGBM	1	1	1
	Persistence time	xgboost	0.5	0.5	0.571428571
		random forest	0.5	0.5	0.571428571

Table S7. SHAP score values of different determinants based on XGBoost.

		airline	climate	socioeconomic	population	forest
Source	DENV-1	0.15	0.37	0.10	0.35	0.03
	DENV-2	0.30	0.34	0.13	0.22	0.01
	DENV-3	0.27	0.13	0.30	0.29	0.01
	DENV-4	0.46	0.18	0.11	0.14	0.10
Hub	DENV-1	0.24	0.15	0.15	0.25	0.20
	DENV-2	0.35	0.49	0.06	0.07	0.03
	DENV-3	0.19	0.52	0.05	0.15	0.09
	DENV-4	0.12	0.61	0.12	0.10	0.05
Destination	DENV-1	0.22	0.24	0.15	0.32	0.07
	DENV-2	0.21	0.67	0.05	0.02	0.05
	DENV-3	0.11	0.10	0.12	0.51	0.16
	DENV-4	0.11	0.25	0.11	0.11	0.43
Persistence	DENV-1	0.44	0.09	0.09	0.32	0.07
	DENV-2	0.43	0.09	0.26	0.17	0.06
	DENV-3	0.73	0.00	0.07	0.12	0.08
	DENV-4	0.43	0.00	0.00	0.52	0.05

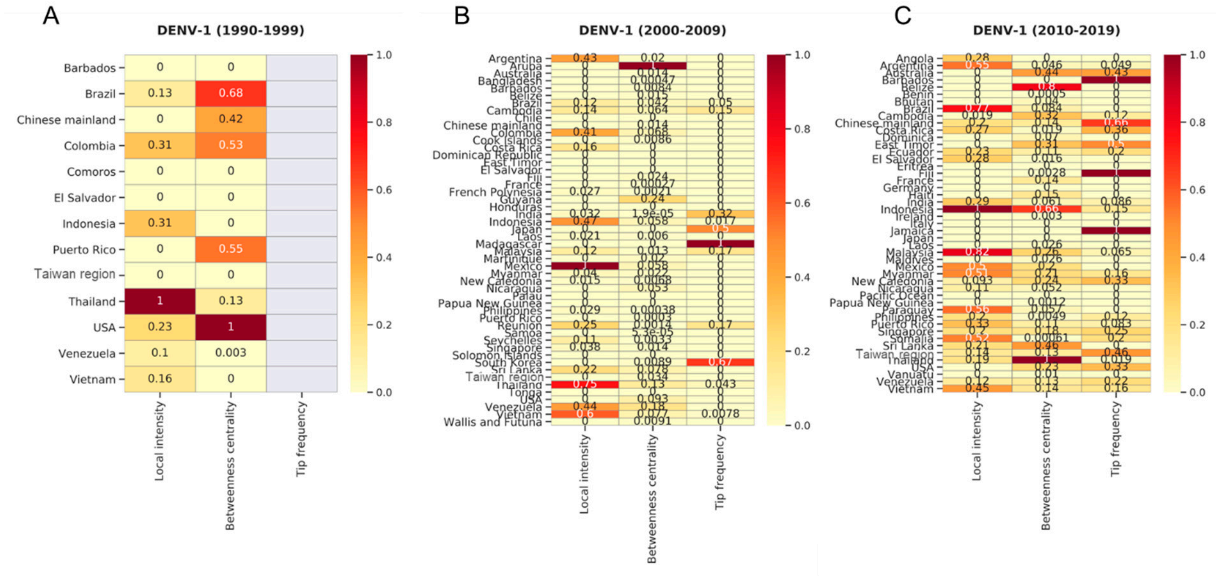

Figure S1: Three circulation indicators in three decades of DENV-1. The horizontal axis is the three indicators and the vertical axis is the countries or regions. The three decades during 1990-2019 were showed in Figure A, B and C, respectively. The range of the normalized value of local intensity, betweenness centrality and tip frequency is 0-1. The gray grid represents the indicator is unavailable.

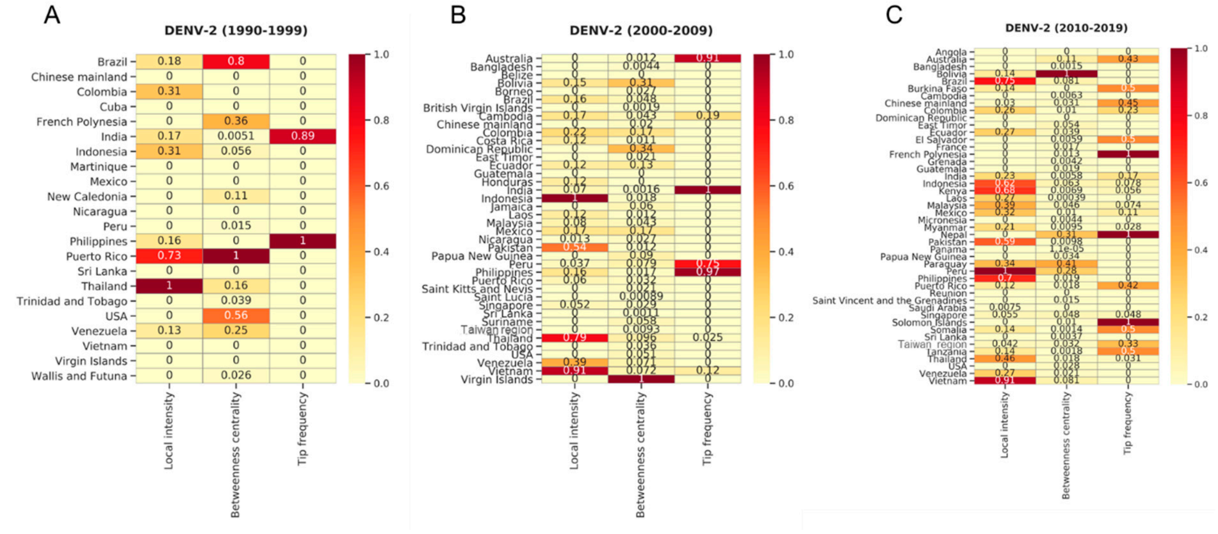

Figure S2: Three circulation indicators in three decades of DENV-2. The horizontal axis is the three indicators and the vertical axis is the countries or regions. The three decades during 1990-2019 were showed in Figure A, B and C, respectively. The range of the normalized value of local intensity, betweenness centrality and tip frequency is 0-1 and persistence time is displayed as absolute numbers.

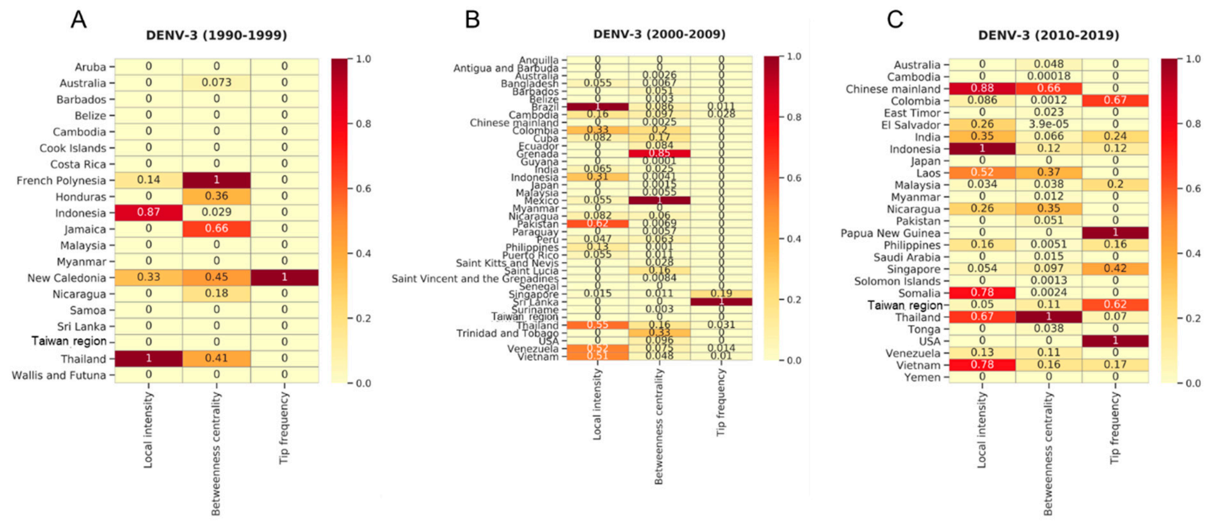

Figure S3: Three circulation indicators in three decades of DENV-3. The horizontal axis is the three indicators and the vertical axis is the countries or regions. The three decades during 1990-2019 were showed in Figure A, B and C, respectively. The range of the normalized value of local intensity, betweenness centrality and tip frequency is 0-1 and persistence time is displayed as absolute numbers.

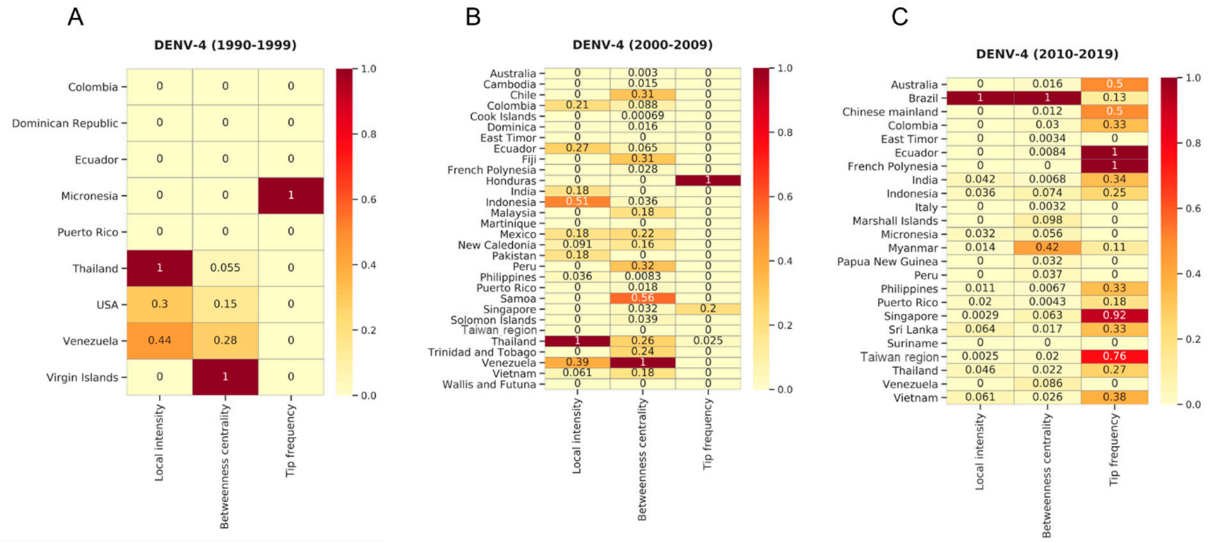

Figure S4: Three circulation indicators in three decades of DENV-4. The horizontal axis is the three indicators and the vertical axis is the countries or regions. The three decades during 1990-2019 were showed in Figure A, B and C, respectively. The range of the normalized value of local intensity, betweenness centrality and tip frequency is 0-1.

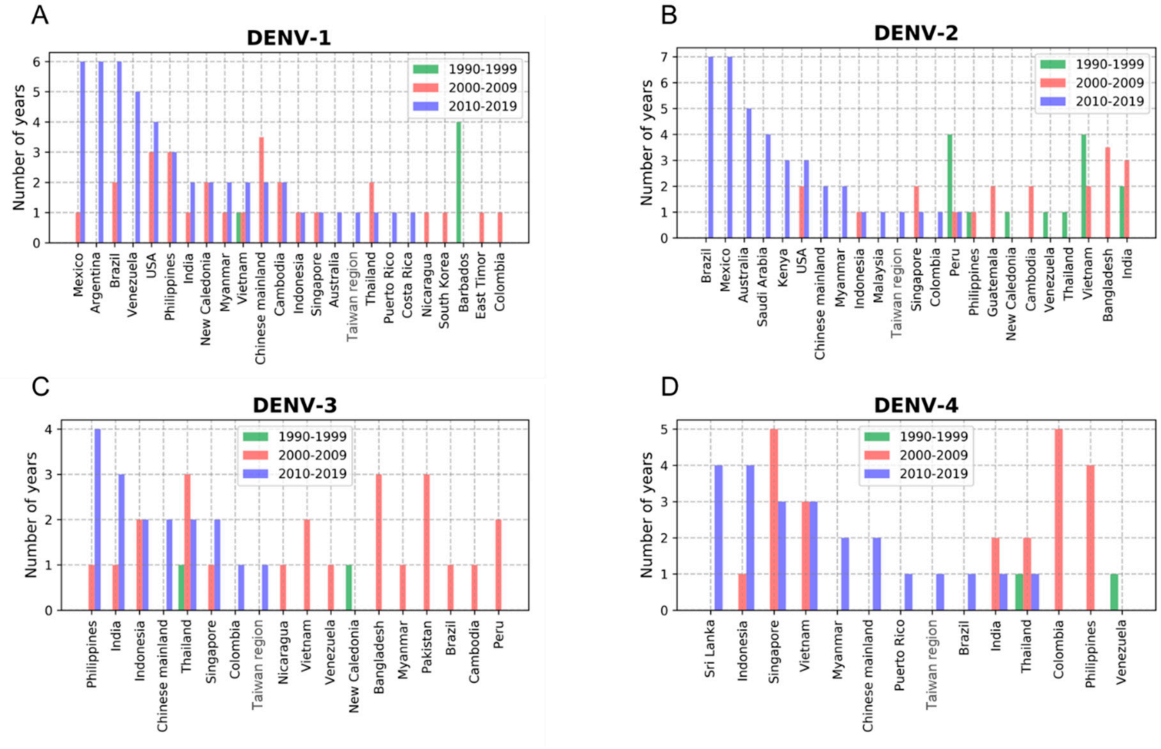

Figure S5: Median persistence time in different regions for three decades. Figure A, B, C and D show the results of DENV-1, 2, 3 and 4, respectively. The horizontal axis is the countries or regions and the vertical axis is the number of years. 1990-1999, 2000-2009 and 2010-2019 are displayed with green, red and blue, respectively.

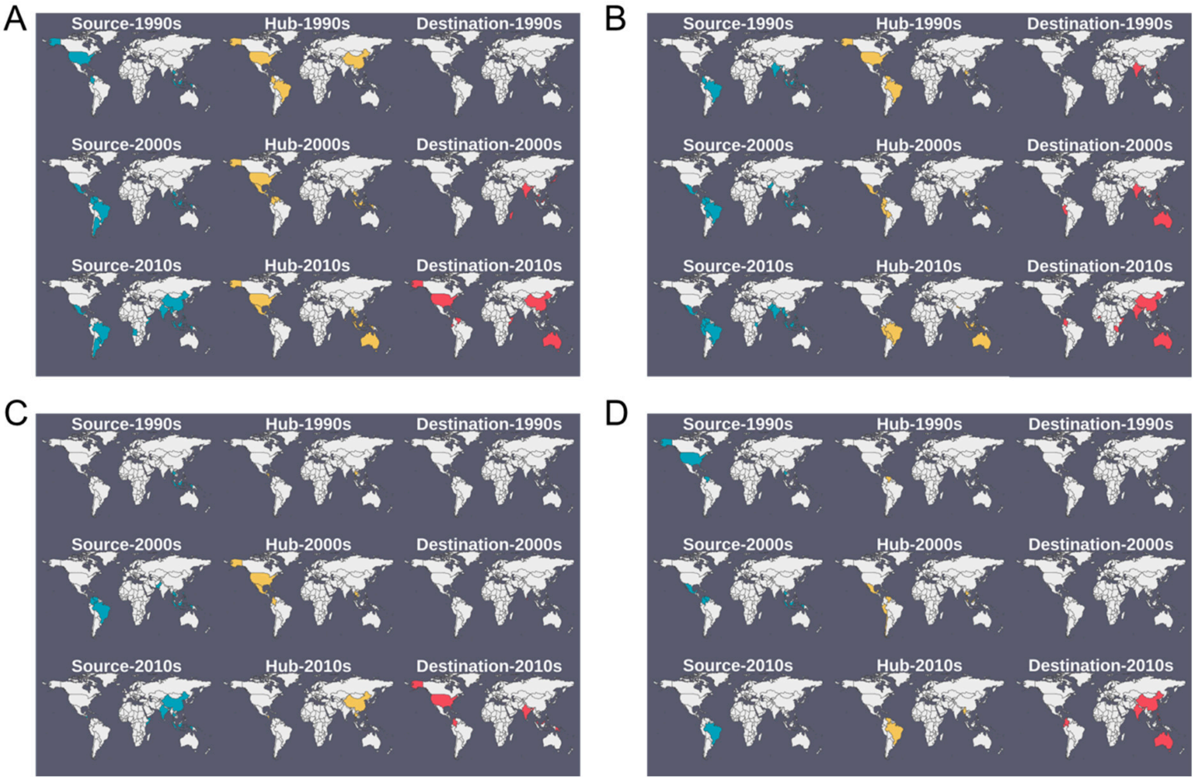

Figure S6: Spatial distribution of different circulation roles during 1990-2019. Figure A, B, C and

D show the results of DENV-1, 2, 3 and 4, respectively. The colors of teal, orange and red represent source, hub and destination, respectively. In each figure, the first, second and third rows are the distribution in the decade of 1990-1999, 2000-2009 and 2010-2019, respectively.

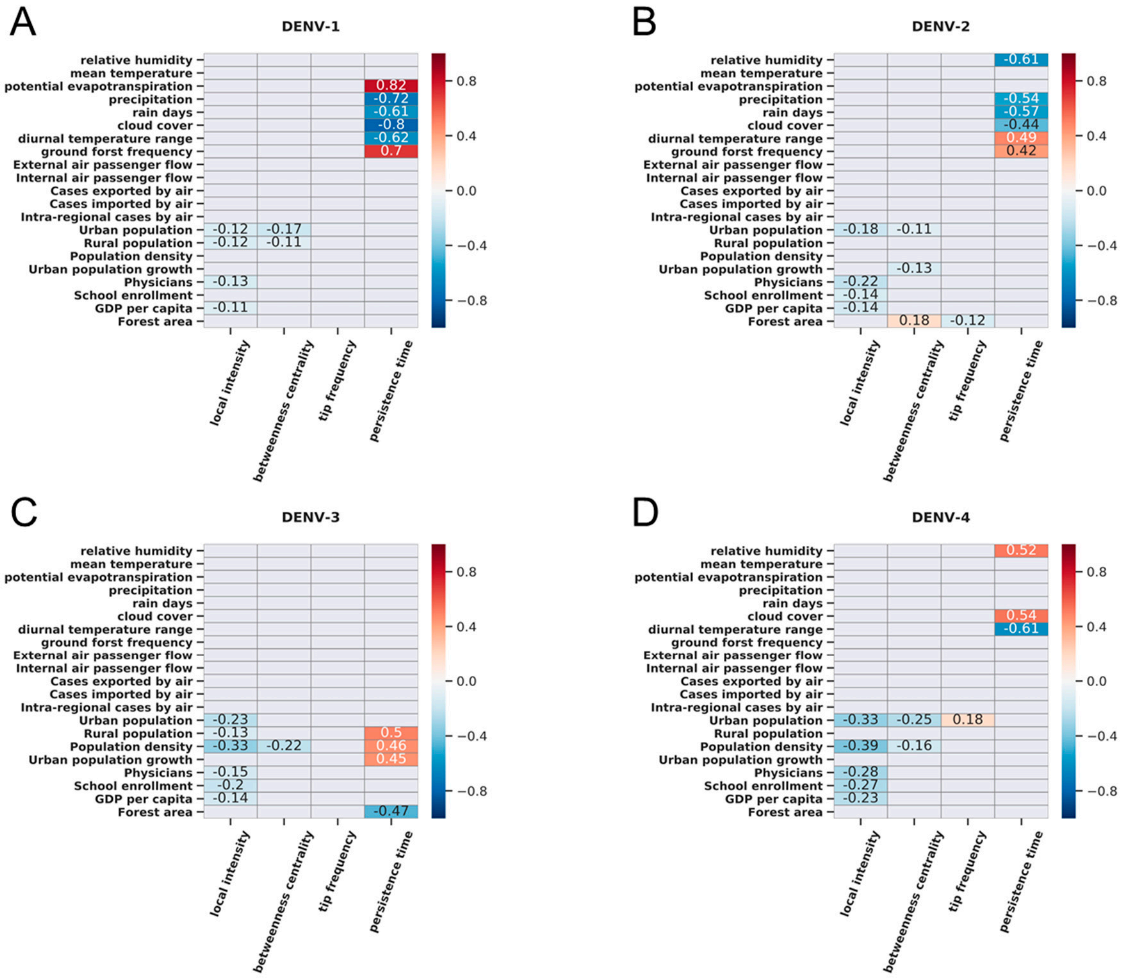

Figure S7: Spearman's coefficient between the four indicators and factors. Figure A, B, C and D show the results of DENV-1, 2, 3 and 4, respectively. The horizontal axis is the four circulation indicators and the vertical axis is the factors. Values in the grid are statistically significant results ($p<0.05$) and gray grids represents non-significant results.

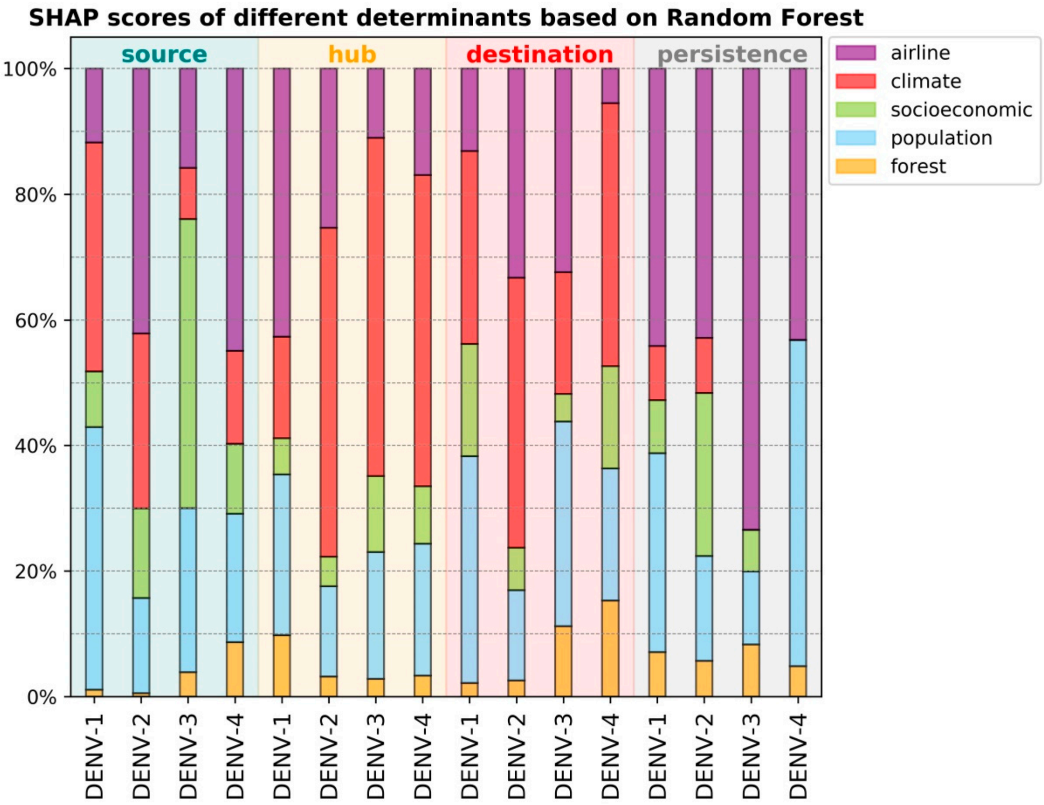

Figure S8: SHAP scores of different determinants based on Random Forest. The horizontal axis is the four serotypes and the vertical axis is the proportion of SHAP scores of different determinants. Five categories of the determinants, including climate, airline, population, socioeconomic and forest factors, are colored as showed in the right legend. Results for the source, hub, destination, and persistence are colored by teal, orange, red and grey respectively.

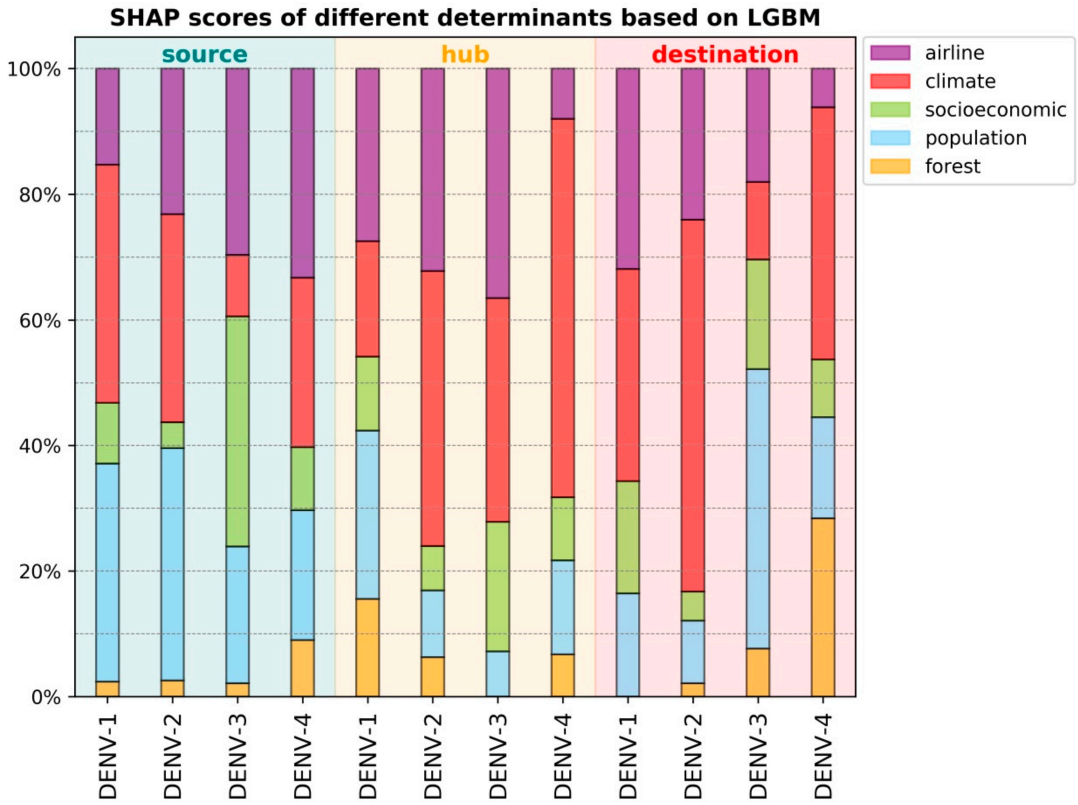

Figure S9: SHAP scores of different determinants based on LGBM. The horizontal axis is the four serotypes and the vertical axis is the proportion of SHAP scores of different determinants. Five categories of the determinants, including climate, airline, population, socioeconomic and forest factors, are colored as showed in the right legend. Results for the source, hub, and destination are colored by teal, orange, and red respectively.

The mathematical expression of SHAP (SHapley Additive exPlanation) is as:

$$g(z') = \Phi_0 + \sum_{j=1}^{N} \Phi_j$$

$g(z')$ denotes the explanatory model. N represents the number of input features. Φ_j denotes the SHAP value of each feature and Φ_0 is a constant. For each prediction sample, the model $g(z')$ calculates a prediction value and the SHAP value represents the contribution of each feature in the sample.

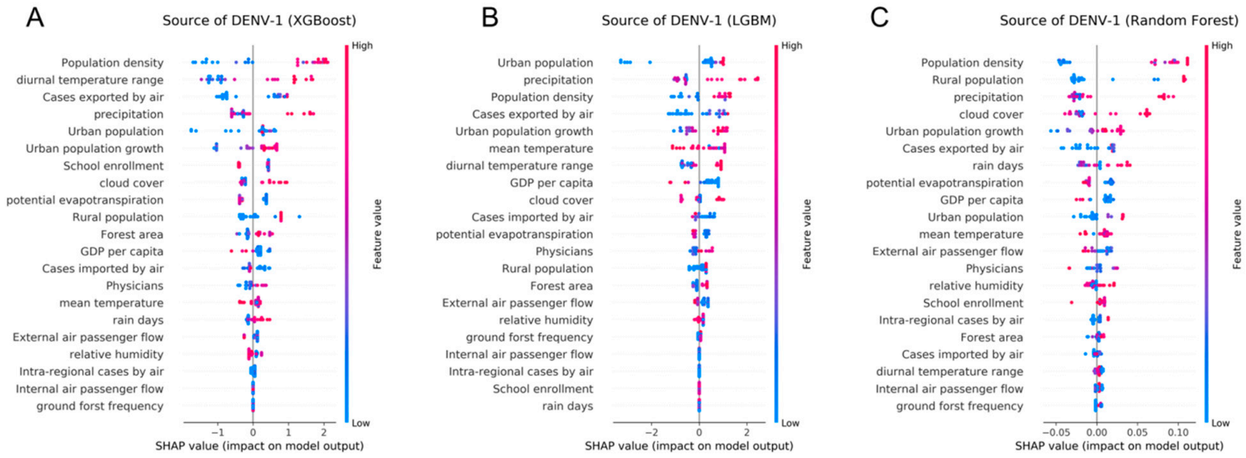

Figure S10: SHAP values of features to classify source of DENV-1. Figure A is the results identified by XGBoost and Figure B by LGBM. The horizontal axis is the SHAP values and the vertical axis is the ranking 21 features based on importance. Colors from blue to red indicate values of the features from low to high.

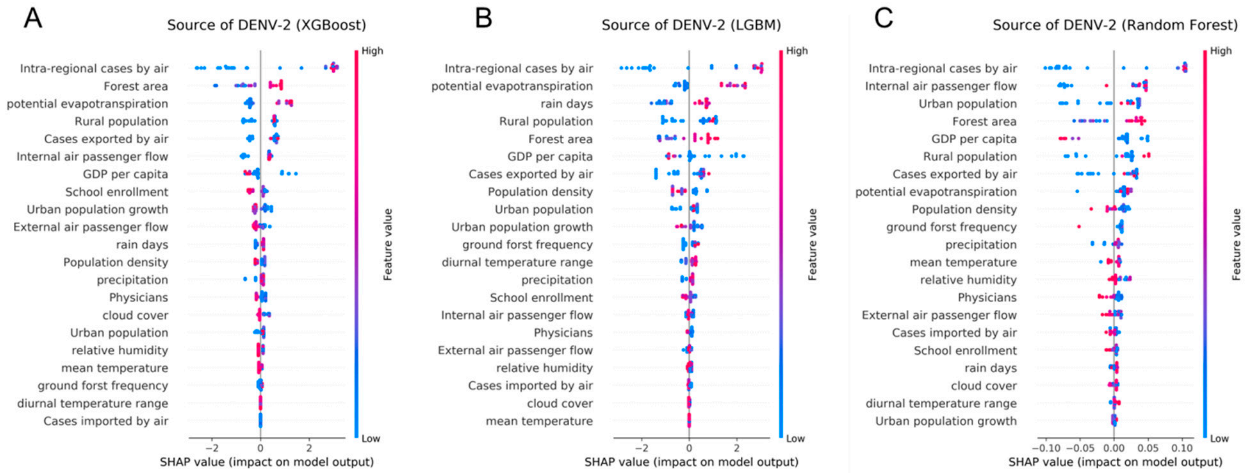

Figure S11: SHAP values of features to classify source of DENV-2. Figure A is the results identified by XGBoost and Figure B by LGBM. The horizontal axis is the SHAP values and the vertical axis is the ranking 21 features based on importance. Colors from blue to red indicate values of the features from low to high.

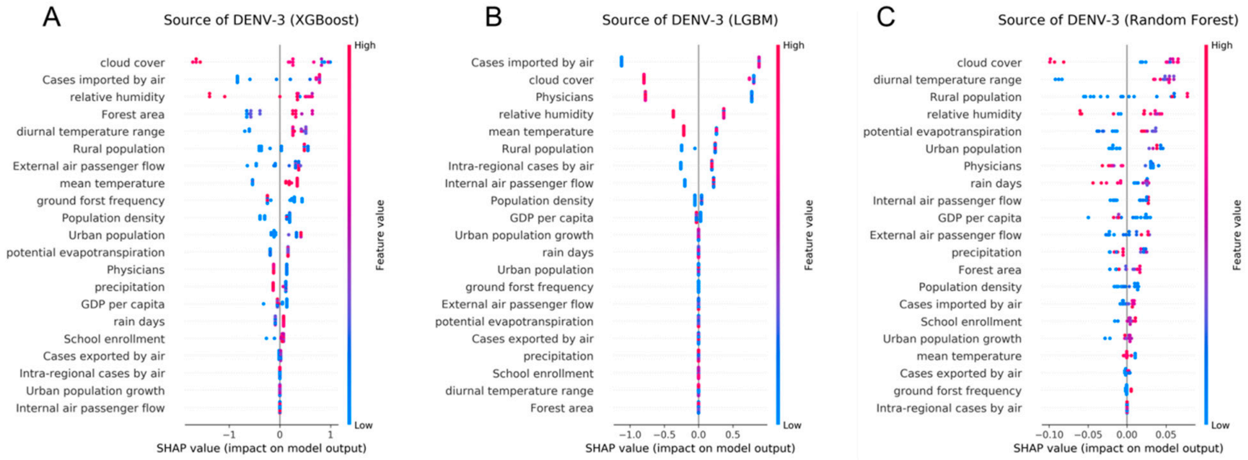

Figure S12: SHAP values of features to classify source of DENV-3. Figure A is the results identified by XGBoost and Figure B by LGBM. The horizontal axis is the SHAP values and the vertical axis is the ranking 21 features based on importance. Colors from blue to red indicate values of the features from low to high.

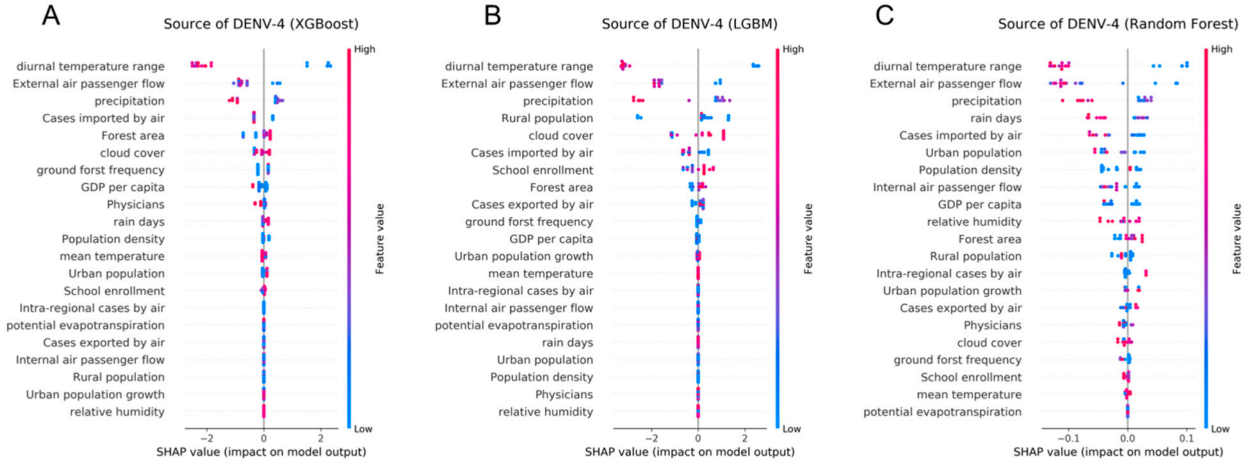

Figure S13: SHAP values of features to classify source of DENV-4. Figure A is the results identified by XGBoost and Figure B by LGBM. The horizontal axis is the SHAP values and the vertical axis is the ranking 21 features based on importance. Colors from blue to red indicate values of the features from low to high.

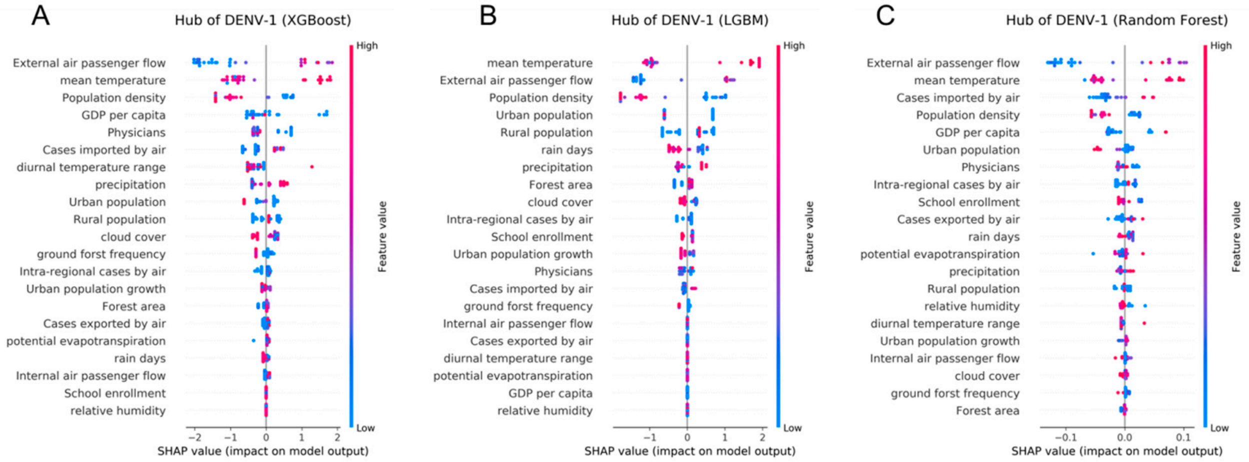

Figure S14: SHAP values of features to classify hub of DENV-1. Figure A is the results identified by XGBoost and Figure B by LGBM. The horizontal axis is the SHAP values and the vertical axis is the ranking 21 features based on importance. Colors from blue to red indicate values of the features from low to high.

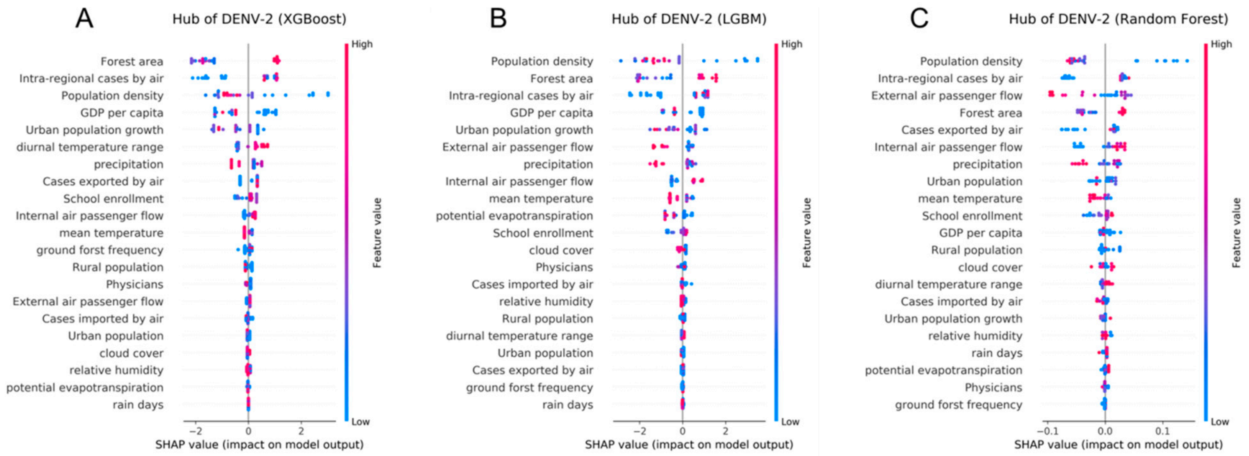

Figure S15: SHAP values of features to classify hub of DENV-2. Figure A is the results identified by XGBoost and Figure B by LGBM. The horizontal axis is the SHAP values and the vertical axis is the ranking 21 features based on importance. Colors from blue to red indicate values of the features from low to high.

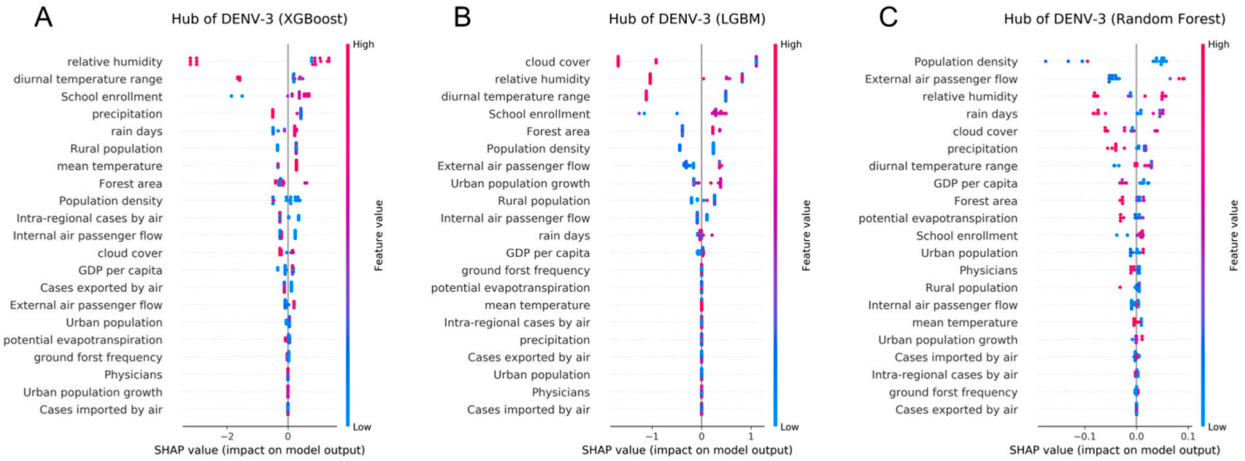

Figure S16: SHAP values of features to classify hub of DENV-3. Figure A is the results identified by XGBoost and Figure B by LGBM. The horizontal axis is the SHAP values and the vertical axis is the ranking 21 features based on importance. Colors from blue to red indicate values of the features from low to high.

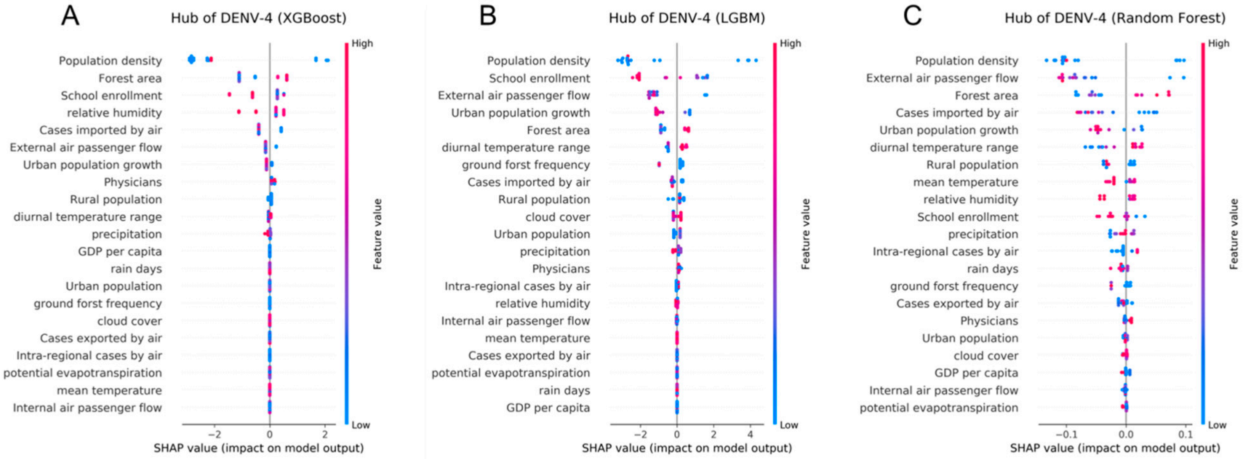

Figure S17: SHAP values of features to classify hub of DENV-4. Figure A is the results identified by XGBoost and Figure B by LGBM. The horizontal axis is the SHAP values and the vertical axis is the ranking 21 features based on importance. Colors from blue to red indicate values of the features from low to high.

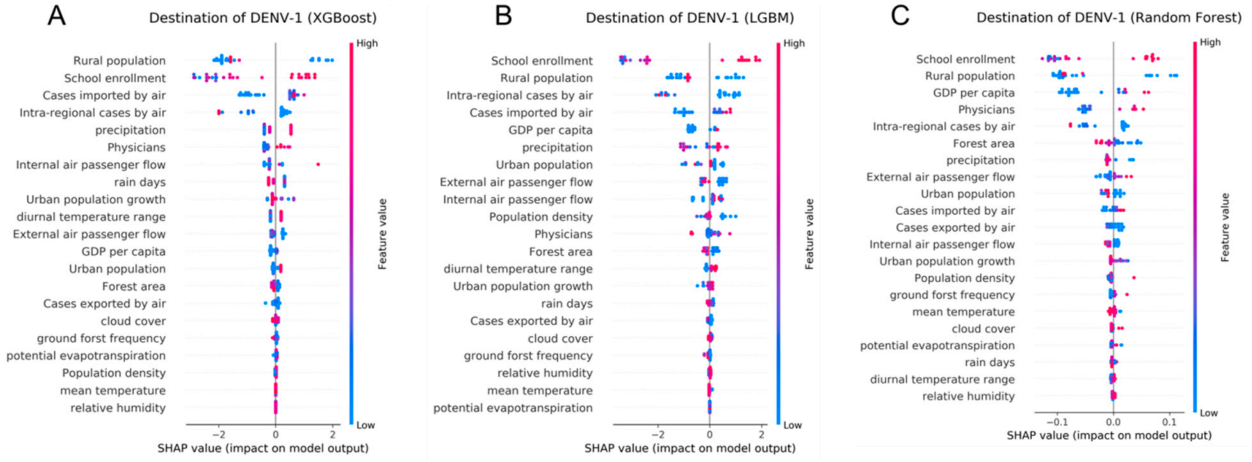

Figure S18: SHAP values of features to classify destination of DENV-1. Figure A is the results identified by XGBoost and Figure B by LGBM. The horizontal axis is the SHAP values and the vertical axis is the ranking 21 features based on importance. Colors from blue to red indicate values of the features from low to high.

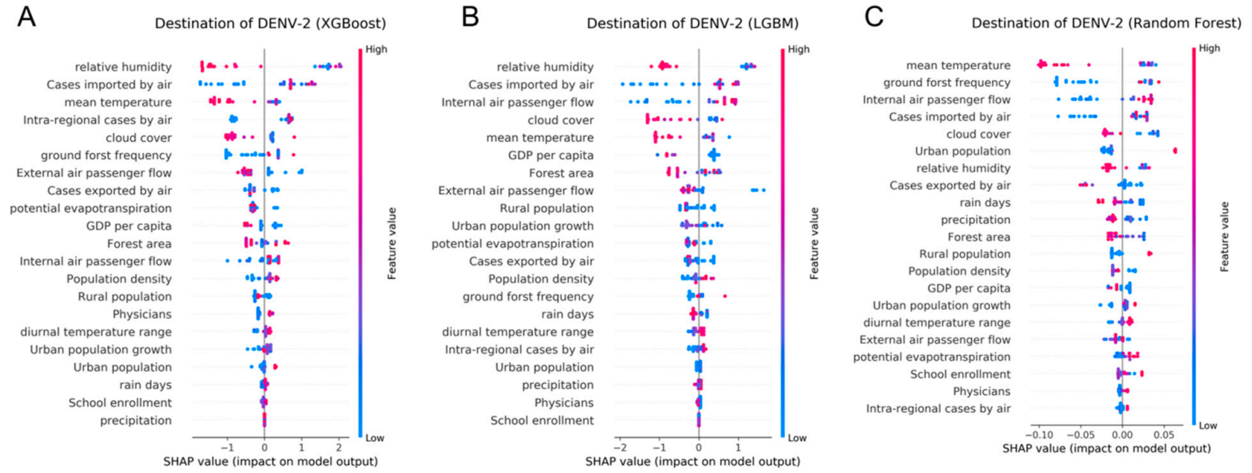

Figure S19: SHAP values of features to classify destination of DENV-2. Figure A is the results identified by XGBoost and Figure B by LGBM. The horizontal axis is the SHAP values and the vertical axis is the ranking 21 features based on importance. Colors from blue to red indicate values of the features from low to high.

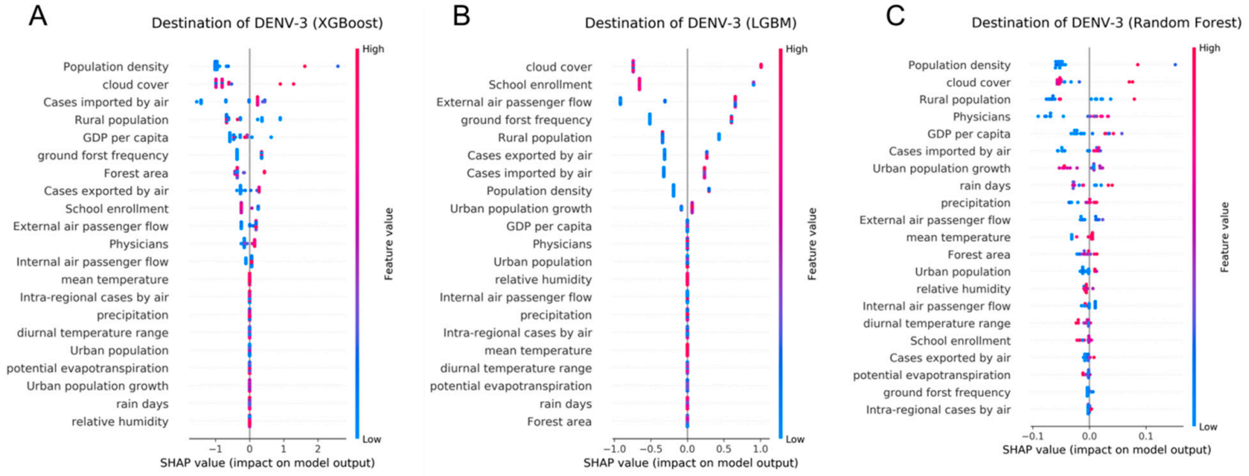

Figure S20: SHAP values of features to classify destination of DENV-3. Figure A is the results identified by XGBoost and Figure B by LGBM. The horizontal axis is the SHAP values and the vertical axis is the ranking 21 features based on importance. Colors from blue to red indicate values of the features from low to high.

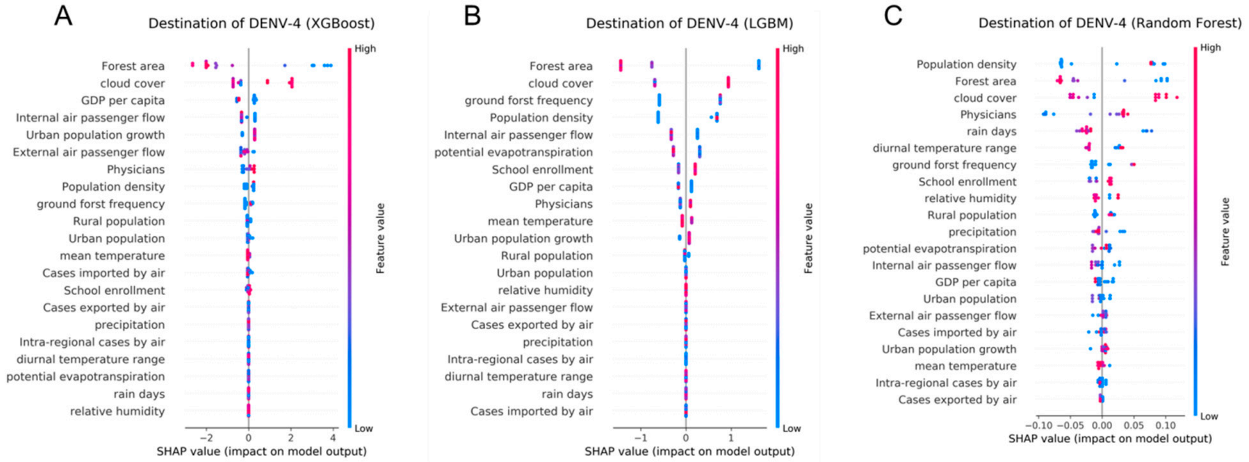

Figure S21: SHAP values of features to classify destination of DENV-4. Figure A is the results identified by XGBoost and Figure B by LGBM. The horizontal axis is the SHAP values and the vertical axis is the ranking 21 features based on importance. Colors from blue to red indicate values of the features from low to high.

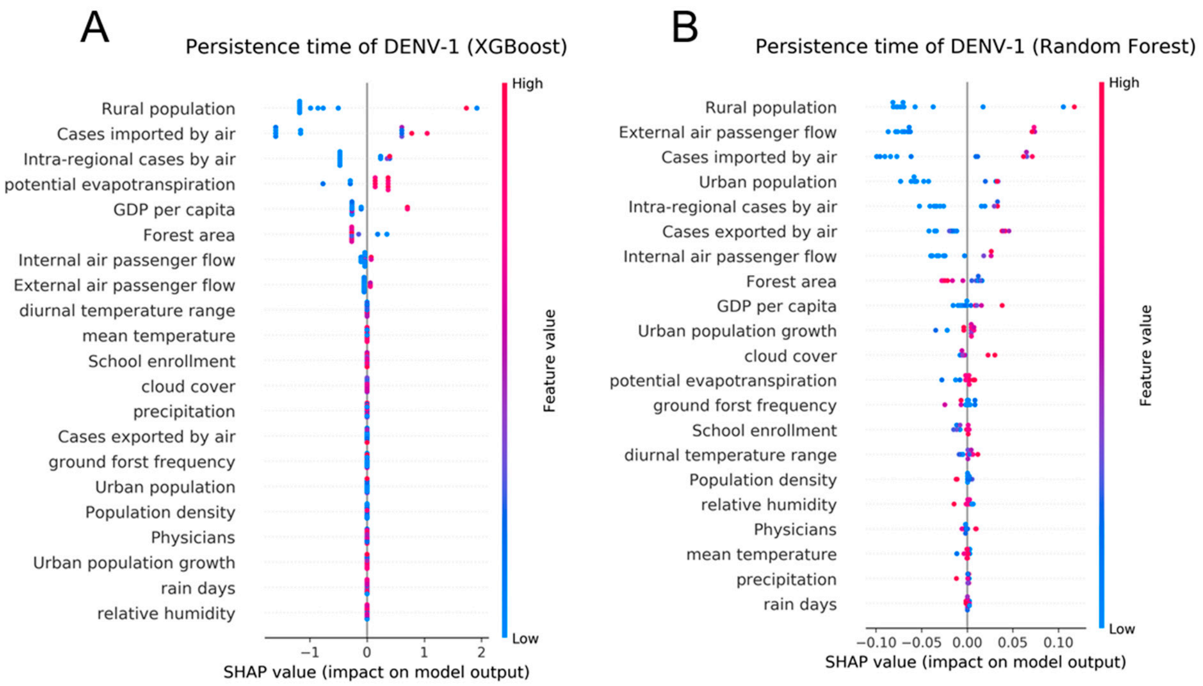

Figure S22: SHAP values of features to determine persistence time of DENV-1. Figure A is the results identified by XGBoost and Figure B by LGBM. The horizontal axis is the SHAP values and the vertical axis is the ranking 21 features based on importance. Colors from blue to red indicate values of the features from low to high.

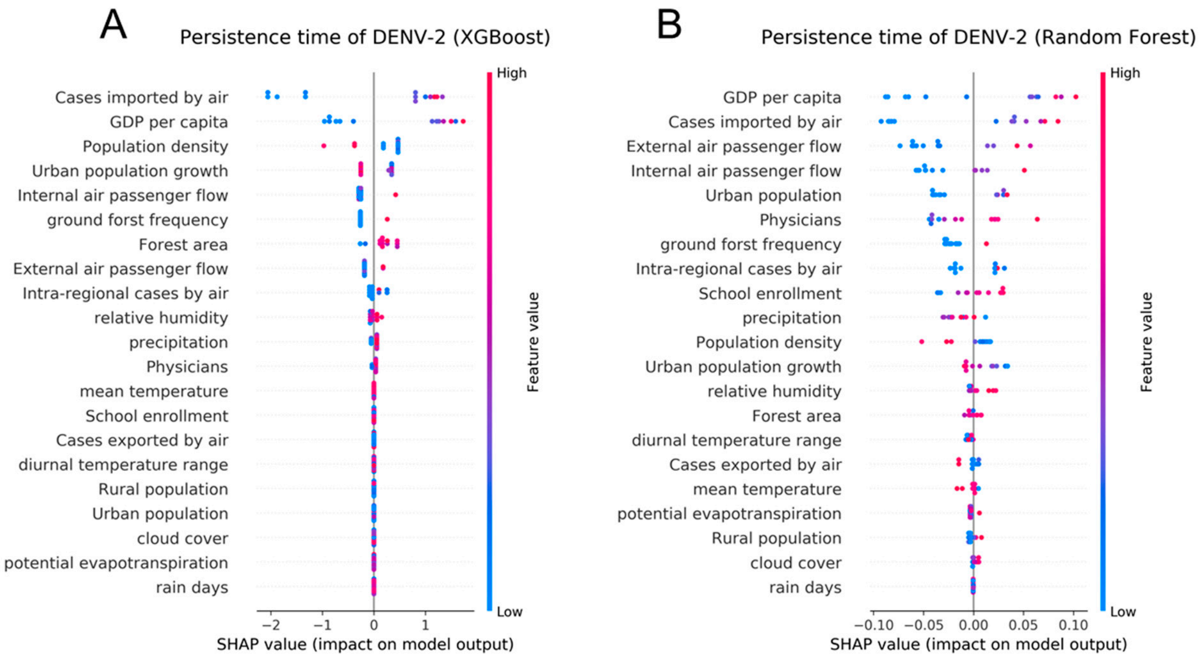

Figure S23: SHAP values of features to determine persistence time of DENV-2. Figure A is the results identified by XGBoost and Figure B by LGBM. The horizontal axis is the SHAP values and the vertical axis is the ranking 21 features based on importance. Colors from blue to red indicate values of the features from low to high.

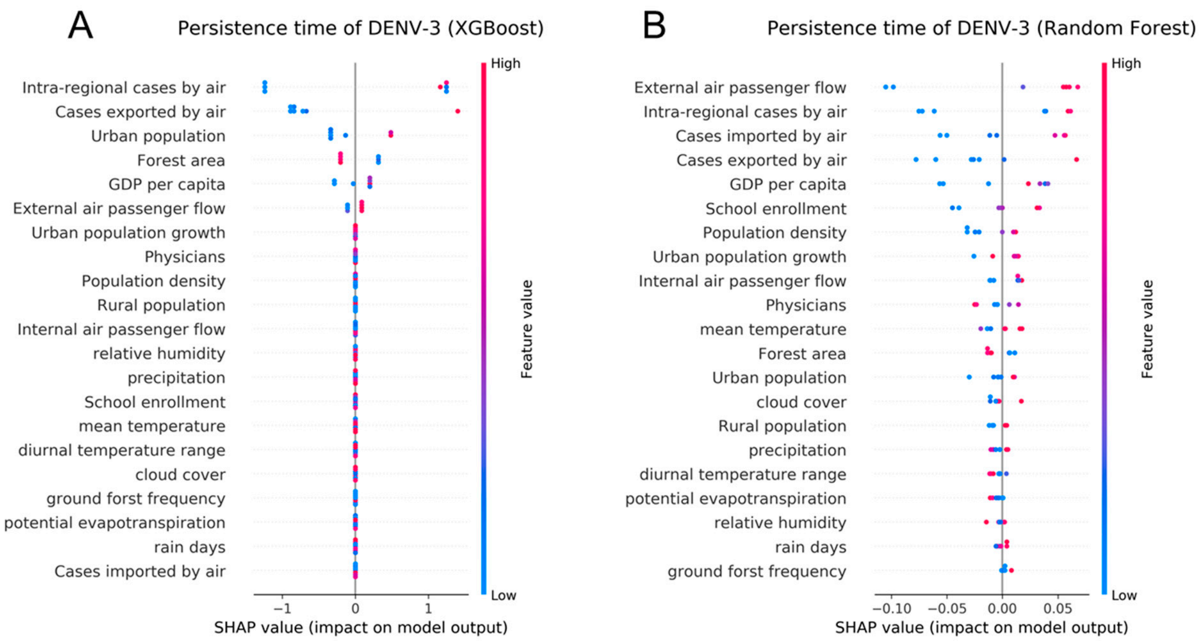

Figure S24: SHAP values of features to determine persistence time of DENV-3. Figure A is the results identified by XGBoost and Figure B by LGBM. The horizontal axis is the SHAP values and the vertical axis is the ranking 21 features based on importance. Colors from blue to red indicate values of the features from low to high.

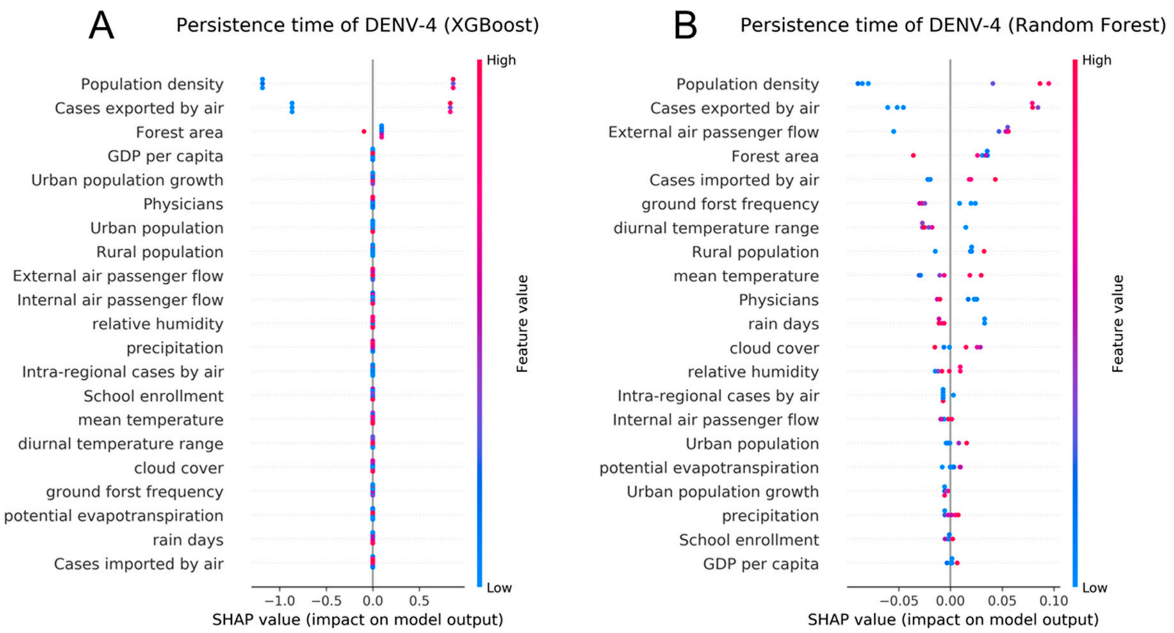

Figure S25: SHAP values of features to determine persistence time of DENV-4. Figure A is the results identified by XGBoost and Figure B by LGBM. The horizontal axis is the SHAP values and the vertical axis is the ranking 21 features based on importance. Colors from blue to red indicate values of the features from low to high.
